# Characterizing Spatiotemporal Population Receptive Fields in Human Visual Cortex with fMRI

**DOI:** 10.1523/JNEUROSCI.0803-23.2023

**Published:** 2024-01-10

**Authors:** Insub Kim, Eline R. Kupers, Garikoitz Lerma-Usabiaga, Kalanit Grill-Spector

**Affiliations:** ^1^ Department of Psychology, Stanford University, Stanford, CA, 94305; ^2^ BCBL. Basque Center on Cognition, Brain and Language, 20009 San Sebastian, Spain; ^3^ IKERBASQUE. Basque Foundation for Science, 48009 Bilbao, Spain; ^4^ Wu Tsai Neurosciences Institute, Stanford University, Stanford, CA, 94305

**Keywords:** fMRI, human visual cortex, pRF, spatiotemporal

## Abstract

The use of fMRI and computational modeling has advanced understanding of spatial characteristics of population receptive fields (pRFs) in human visual cortex. However, we know relatively little about the spatiotemporal characteristics of pRFs because neurons' temporal properties are one to two orders of magnitude faster than fMRI BOLD responses. Here, we developed an image-computable framework to estimate spatiotemporal pRFs from fMRI data. First, we developed a simulation software that predicts fMRI responses to a time-varying visual input given a spatiotemporal pRF model and solves the model parameters. The simulator revealed that ground-truth spatiotemporal parameters can be accurately recovered at the millisecond resolution from synthesized fMRI responses. Then, using fMRI and a novel stimulus paradigm, we mapped spatiotemporal pRFs in individual voxels across human visual cortex in 10 participants (both females and males). We find that a compressive spatiotemporal (CST) pRF model better explains fMRI responses than a conventional spatial pRF model across visual areas spanning the dorsal, lateral, and ventral streams. Further, we find three organizational principles of spatiotemporal pRFs: (1) from early to later areas within a visual stream, spatial and temporal windows of pRFs progressively increase in size and show greater compressive nonlinearities, (2) later visual areas show diverging spatial and temporal windows across streams, and (3) within early visual areas (V1–V3), both spatial and temporal windows systematically increase with eccentricity. Together, this computational framework and empirical results open exciting new possibilities for modeling and measuring fine-grained spatiotemporal dynamics of neural responses using fMRI.

## Significance Statement

We developed a computational framework for estimating spatiotemporal receptive fields of neural populations using fMRI. This framework pushes the boundary of fMRI measurements, enabling quantitative evaluation of neural spatial and temporal processing windows at the resolution of visual degrees and milliseconds, which was thought to be unattainable with fMRI. We not only replicate well-established visual field and population receptive field size maps, but also estimate temporal windows from electrophysiology and electrocorticography. Notably, we find that spatial and temporal windows as well as compressive nonlinearities progressively increase from early to later visual areas in multiple visual processing streams. Together, this framework opens exciting new possibilities for modeling and measuring fine-grained spatiotemporal dynamics of neural responses in the human brain using fMRI.

## Introduction

The visual scene changes over space and time. To interpret this rich visual input, the visual system processes information spatially and temporally through computations by receptive fields. Prior research has separately characterized spatial receptive fields in primate (Hubel and Wiesel, 1968) and human visual cortex (Dumoulin and Wandell, 2008; Wandell et al., 2009; Kay et al., 2013, 2015; Wandell and Winawer, 2015; Klink et al., 2021) as well as temporal properties of neural responses in primates (Maunsell and Gibson, 1992; Nowak and Bullier, 1997) and humans (Horiguchi et al., 2009; Stigliani et al., 2017, 2019; Zhou et al., 2018, 2019; Chai et al., 2019; Harvey et al., 2020; Groen et al., 2022; Hendrikx et al., 2022). However, how spatiotemporal information is jointly processed by receptive fields is not well understood beyond the lateral genicular nucleus (DeSimone and Schneider, 2019), primary visual cortex, V1 (McLean and Palmer, 1989; DeAngelis et al., 1993; De Valois and Cottaris, 1998; De Valois et al., 2000; Conway and Livingstone, 2003; Nishimoto et al., 2011), and motion-selective areas, MT/MST (Simoncelli and Heeger, 1998; Nishimoto and Gallant, 2011; Mineault et al., 2012; Pawar et al., 2019). Thus, it is unknown what are the characteristics of spatiotemporal population receptive fields (pRFs) across human visual cortex.

There are two main reasons for this gap in knowledge. First, measurements of pRFs in humans are derived from fMRI, which typically measures BOLD signals whose timescale is approximately two orders of magnitude slower than the timescale of neural responses (tens to hundreds of milliseconds). Second, there is no integrated framework for mapping and quantifying spatiotemporal pRFs.

**Figure 1.**
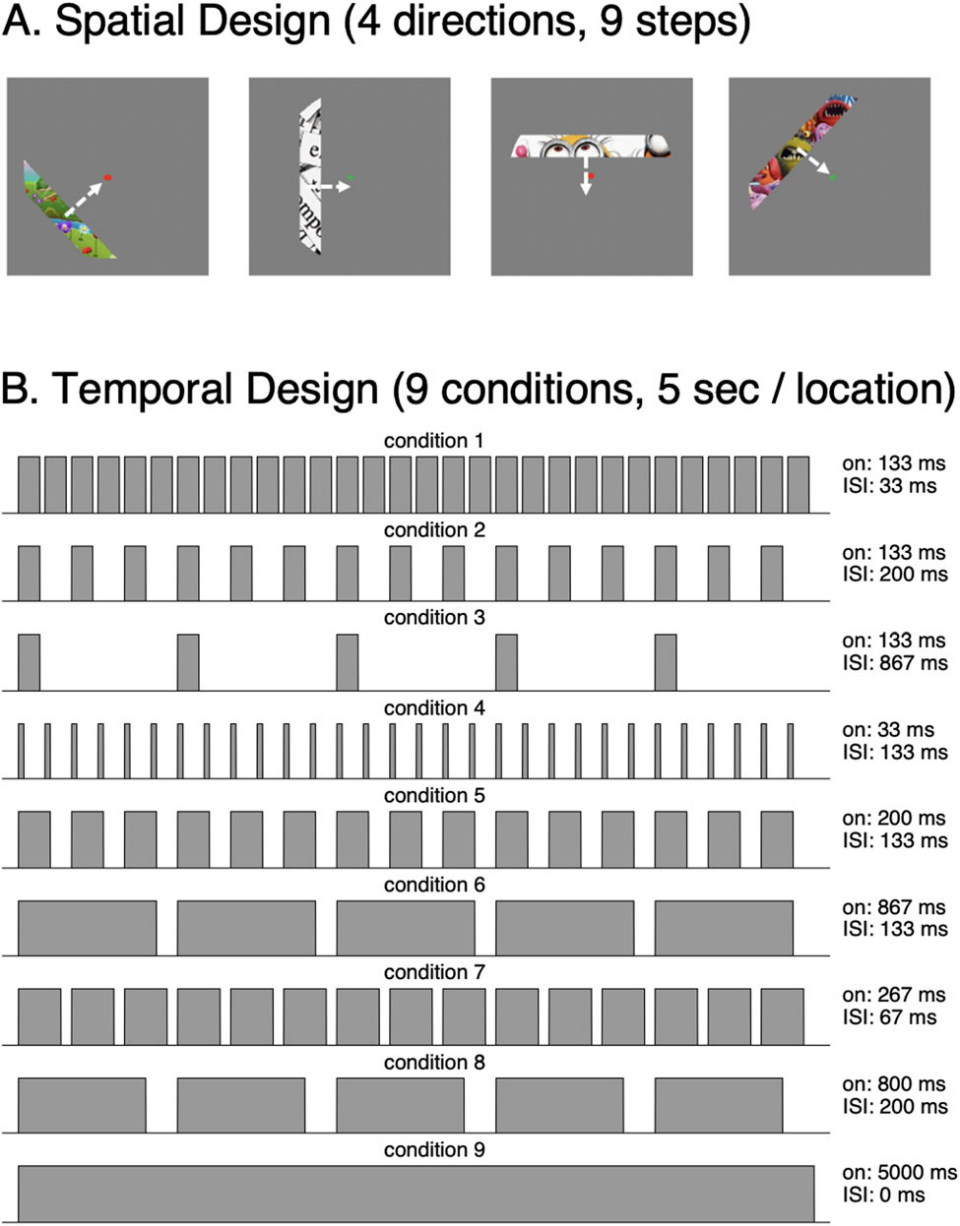
Spatiotemporal pRF experiment. In the experiment, participants viewed a flickering bar that swept the visual field while fixating and performing a color-change task at fixation. ***A***, Spatial design. A bar containing colorful stimuli continuously swept the visual field in four directions and each direction had nine steps. Stimuli swept across a radius of 12° from fixation. Content of the bar was updated with specific timings determined by the temporal condition. ***B***, Temporal design. At each spatial location, the bar was presented for 5 s, in one of nine different temporal conditions shown, in which the bar's content was updated with different random colorful cartoon snippets according to the temporal design of that condition. Each temporal condition was shown in each of the stimulus locations. *On*: on duration for each stimulus presentation. *ISI*: interstimulus interval. Example run: https://github.com/VPNL/stPRF#experiment

Here, we filled this gap in knowledge by developing a method for estimating spatiotemporal pRFs from fMRI. We combined a pRF mapping approach (Dumoulin and Wandell, 2008; Kay et al., 2013) with recent neural temporal encoding approaches (Stigliani et al., 2017, 2019; Zhou et al., 2018, 2019) to estimate spatiotemporal pRFs in each voxel in the visual system. To achieve the desired temporal estimates, we leveraged insights from recent fMRI studies that showed that not only stimulus duration, but also the number of transients and interstimulus intervals (ISIs) produce strong modulation of the amplitude of fMRI signals. To measure spatiotemporal pRFs, we measured each voxel's response to visual stimuli presented in different locations in the visual field under varying presentation timings (Fig. 1). Then, we used a computational framework to estimate spatiotemporal pRF parameters in visual degrees and milliseconds from the fMRI response evoked by the stimulus (Fig. 3). We estimated spatiotemporal pRFs in each voxel of multiple visual areas across three processing streams. The streams emerge in V1, continue to V2 and V3, and diverge into later visual areas in ventral (hV4, VO), lateral (LO, TO), and dorsal (V3AB/IPS) visual cortex.

We examined how characteristics of spatiotemporal pRF may vary across visual areas. One possibility is that spatiotemporal pRFs vary across the visual processing hierarchy. Many studies found that the spatial extent of pRFs progressively increases from early to later visual areas within a processing stream (Larsson and Heeger, 2006; Dumoulin and Wandell, 2008; Kay et al., 2013; Wandell and Winawer, 2015). Additionally, several studies suggest that temporal windows are larger in later than earlier visual areas (Hasson et al., 2008; Honey et al., 2012; Chaudhuri et al., 2015; Baldassano et al., 2017). These findings predict that pRFs in later visual areas will have larger spatial and temporal windows (Zhou et al., 2018). Another possibility is that spatiotemporal pRFs vary across streams. Visual areas in the ventral stream that process static aspects of the stimulus (e.g., VO), may have pRFs with large spatial and large temporal windows (Van Essen and Gallant, 1994). In contrast, areas in the lateral stream that process motion information (e.g., TO), may have spatiotemporal pRFs with large spatial but small temporal windows. These hypotheses are not mutually exclusive, as spatiotemporal pRFs may vary across both stages of the processing hierarchy and stream.

## Materials and Methods

### Participants

The study was approved by the Institutional Review Board of Stanford University. Prior to the start of the study, all participants gave written consent. Ten participants (ages 22–52 years, mean 30.6 years and SD 20.8 years; seven females, three males). The demographics of participants were four East Asian, three White, two multiracial, and one Middle Eastern.

### Spatiotemporal pRF mapping experiment

Participants performed nine runs of the spatiotemporal pRF mapping experiment. While presented with the bar stimuli, participants were instructed to fixate on a central fixation point and performed a color-change detection task. Stimuli consisted of high contrast and colorful cartoon images (Finzi et al., 2021). To elicit a wide range of BOLD response profiles, we systemically varied the location and timing of stimulus presentation. Spatially (Fig. 1*A*), each bar was created by dividing a diagram image (radius of 12° visual angle) into nine distinct apertures. Each bar had a width of 3° visual angle and there was a spatial overlap of 0.375° between adjacent bars. The nine bars corresponded to the nine steps in which the bar swept across the visual field in four different angles (0°, 45°, 90°, and 135°). Temporally (Fig. 1*B*), the duration of each bar location was 5 s. Each 5 s bar location had one of the nine different temporal conditions that varied in duration, ISI, and number of different stimuli. Specifically, temporal conditions 1, 2, and 3 had identical stimulus on durations of 133 ms per image with varying ISIs of 33 ms, 200 ms, and 867 ms, respectively. Temporal conditions 4, 5, and 6 had identical ISIs of 133 ms with varying stimulus on durations of 33 ms, 200 ms, and 867 ms, respectively. Conditions 1, 7, and 8 had an identical total stimulus on duration of 4 s (out of the 5 s in that location) while varying in the number images per bar location: 30, 15, and 5, respectively. Condition 9 had a single stimulus presented for a duration of 5 s without an ISI, which served as a prolonged stimulus condition. The temporal conditions for each location were pseudo-randomly counterbalanced across runs and participants, making each run unique. Across the nine runs, each temporal condition occurred once in each bar location. An example run of the experiment can be viewed on our GitHub repository (https://github.com/VPNL/stPRF#experiment).

### Standard pRF mapping experiment

In a separate session, a traveling wave pRF mapping experiment with cartoon stimuli was conducted to independently define borders of visual regions (Toonotopy; Finzi et al., 2021). Specifically, we defined regions of interest (ROIs) which included: V1, V2, V3, hV4, VO (VO1 and VO2), LO (LO1 and LO2), TO (TO1 and TO2), V3AB, and IPS (IPS0 and IPS1). This pRF mapping experiment used similar stimuli, the same visual field coverage (radius of 12° visual angle), number of angles (0°, 45°, 90°, and 135°), and task (color-change detection task at fixation) as the spatiotemporal pRF mapping experiment. Different from the spatiotemporal pRF experiment: (1) images within each bar consisted of random cartoon images that changed at a constant rate of 8 Hz, (2) bars were spatially less overlapping (0.27°), (3) there were 12 steps in each direction, and (4) each step had a 2-s duration. All participants completed four runs of the Toonotopy experiment.

### fMRI acquisition and preprocessing

During fMRI, stimuli were presented using an Eiki LC-WUL100L projector (resolution: 1,920 × 1,200; refresh rate: 60 Hz) using MATLAB (http://www.mathworks.com/) and Psychophysics Toolbox (Brainard, 1997; http://psychtoolbox.org). MRI scanning was conducted on a 3T scanner (Signa, GE) with a Nova 16-channel head coil. Functional data were acquired using a T2*-weighted gradient-echo echo-planar imaging (GE-EPI) sequence (flip angle = 62°, TR = 1,000 ms, TE = 30 ms, field of view = 192 mm, 2.4 mm isotropic voxel size). Slices were prescribed to be perpendicular to calcarine sulcus to cover occipitotemporal cortex. A T1-weighted inplane image was collected for each participant, using the same prescription as the functional data, but higher resolution (0.75 × 0.75 × 2.4 mm) to aid alignment to anatomical scan. A high-resolution anatomical scan (MPRAGE T1-weighted BRAVO pulse sequence, inversion time = 450 ms, flip angle = 12°, TE = 2.91 ms, 1 mm isotropic voxel size, field of view = 240 × 240 mm) were collected using a Nova 32-channel head coil. This anatomical scan was segmented in white and gray brain matter and used to reconstruct the cortical surface with FreeSurfer [version 6.0; Fischl, 2012 (http://freesurfer.net/)].

Functional data were preprocessed using Vistasoft (http://github.com/vistalab/vistasoft) and SPM12 (https://github.com/spm/spm12). Functional images were aligned to each participant's native space using T1-weighted inplane images. Then, the functional data were motion-corrected, and each voxel's time courses were converted to percent signal change.

### Fitting hemodynamic response functions for individual voxels

It has been well characterized that stimulus-evoked BOLD responses depend on both neuronal and hemodynamic properties (Lindquist and Wager, 2007; Polimeni and Lewis, 2021) and further, hemodynamic responses may vary in response to different types of stimuli, among different regions of the visual cortex, and across individuals (Handwerker et al., 2004). All these factors may contribute to temporal parameter estimates in our model. In other words, if there are systematic variations in hemodynamic response functions (HRFs) across different voxels and brain regions, using a single HRF for analysis may result in inaccurate estimates of spatiotemporal pRF parameters.

Thus, we performed an iterative linear fitting approach to estimate an optimized HRF for each voxel. First, we generated a stimulus design matrix for the spatiotemporal pRF mapping experiment with 36 conditions (one condition for each of the nine bar locations and four orientations). Then, using a general linear model (GLM) approach, this design matrix was convolved with an HRF to generate predictors for each condition. For each iteration and for each voxel, the HRF parameters were optimized to minimize the difference between predicted fMRI time course and fMRI data. HRFs were parameterized as a sum of two-gamma functions (Friston et al., 1998) where each gamma function had two parameters: peak latency and full-width at half-maximum (FWHM). The default Vistasoft HRF was also generated by using the two-gamma functions with peaks of 5.4 and 10.9, and FWHMs of 5.2, 7.35. Critically, the GLM design matrix coded the spatial location and orientation of the bar and disregarded the fine-grained temporal properties of the stimulus. Given that our experimental design included a wide range of temporal variabilities, the estimated HRFs are not biased to a specific temporal stimulus condition.

**Figure 2.**
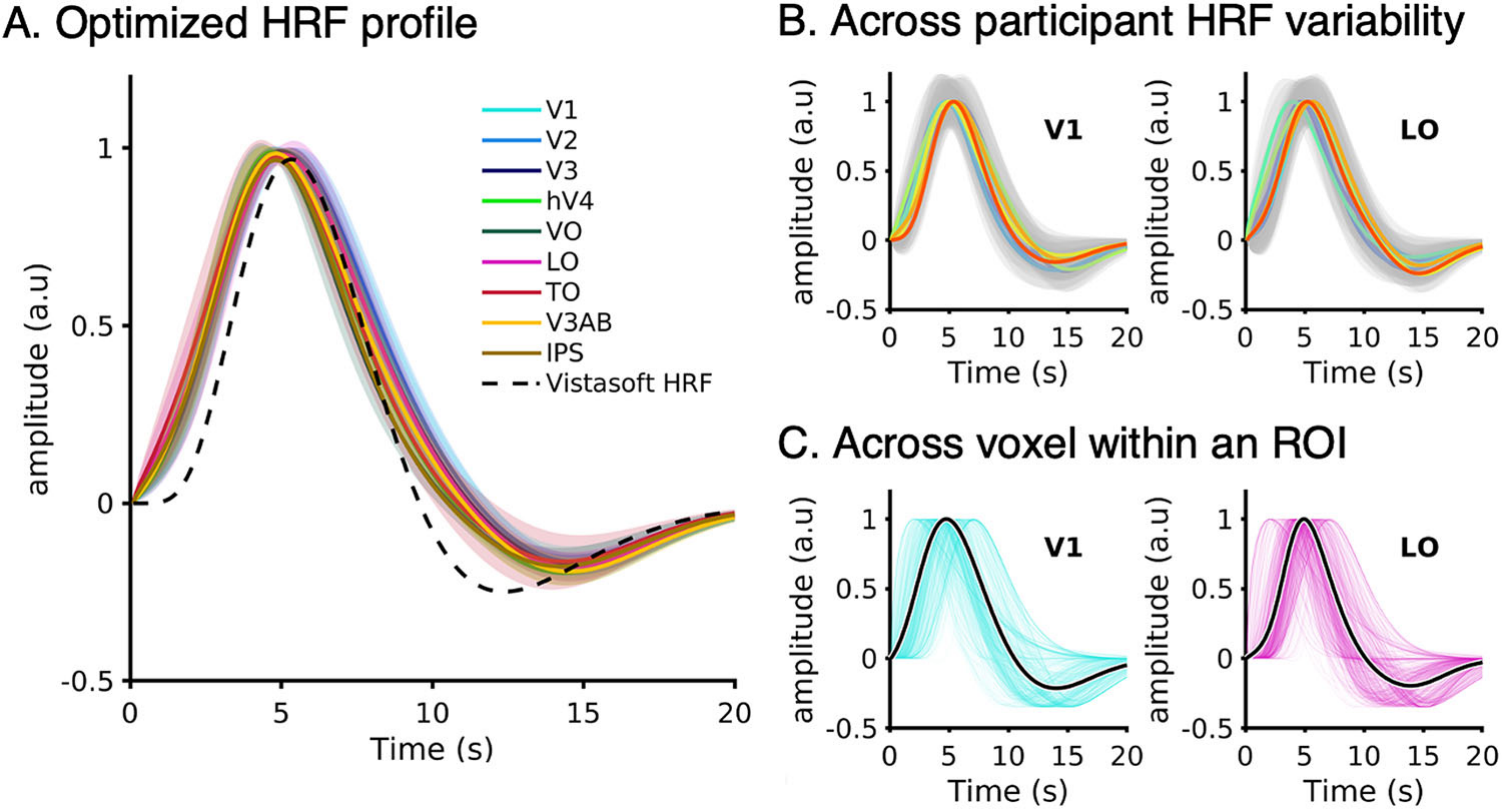
Optimized HRFs for individual voxels. For each voxel, we estimated its HRF using an optimization procedure. ***A***, The average estimated HRF for each visual area. HRFs were averaged across voxels and participants. Heights of the HRFs are normalized to be 1 for visual comparison. *Blues:* V1, V2, and V3; *Greens:* hV4 and VO; *Reds:* LO and TO; *Yellows:* V3AB and IPS. *Shaded areas*: standard deviation across 10 participants. *Dashed line*: default HRF from Vistasoft. ***B***, To illustrate the across-participant HRF variability, the mean HRF for V1 (left) and LO (right) is plotted for each individual participant. Each colored line indicates an individual participant. *Shaded gray area:* standard deviation across voxels for each participant. ***C***, To visualize within ROI variability of HRFs, we show the HRFs of all voxels in V1 (left) and LO (right) from an example participant. Each line indicates the estimated HRF for a single voxel. *Solid black line:* the average HRF across voxels of that visual area.

On average, the optimized HRFs across different visual areas were consistent across participants (Fig. 2*A*,*B*). The estimated HRFs for all visual regions showed similar time-to-peak compared with the Vistasoft HRF (Fig. 2*A*, dashed line), but some differences were observed including an earlier onset, wider width, and delayed undershoot. The across participant variability of estimated average HRFs was small (Fig. 2*B*). However, when examining individual voxels' HRFs within a visual area, we found a large degree of variation. As an example, while the average HRF profile for V1 and LO appeared similar (Fig. 2*C*, black lines), there was substantial variability of HRF across voxels spanning these regions within a single participant (Fig. 2*C*, colored lines). All results reported are with a voxel-wise optimized HRF unless otherwise stated.

### Spatiotemporal pRF modeling framework

The spatiotemporal pRF model is a stimulus-referred encoding model that predicts the BOLD response of each voxel while estimating both spatial and temporal neural characteristics of the pRF given a stimulus sequence. In general, an underlying assumption of the spatiotemporal pRF is that visual neurons integrate stimulus information over visual space and time (Adelson and Bergen, 1985; Watson and Ahumada, 1985).

**Figure 3.**
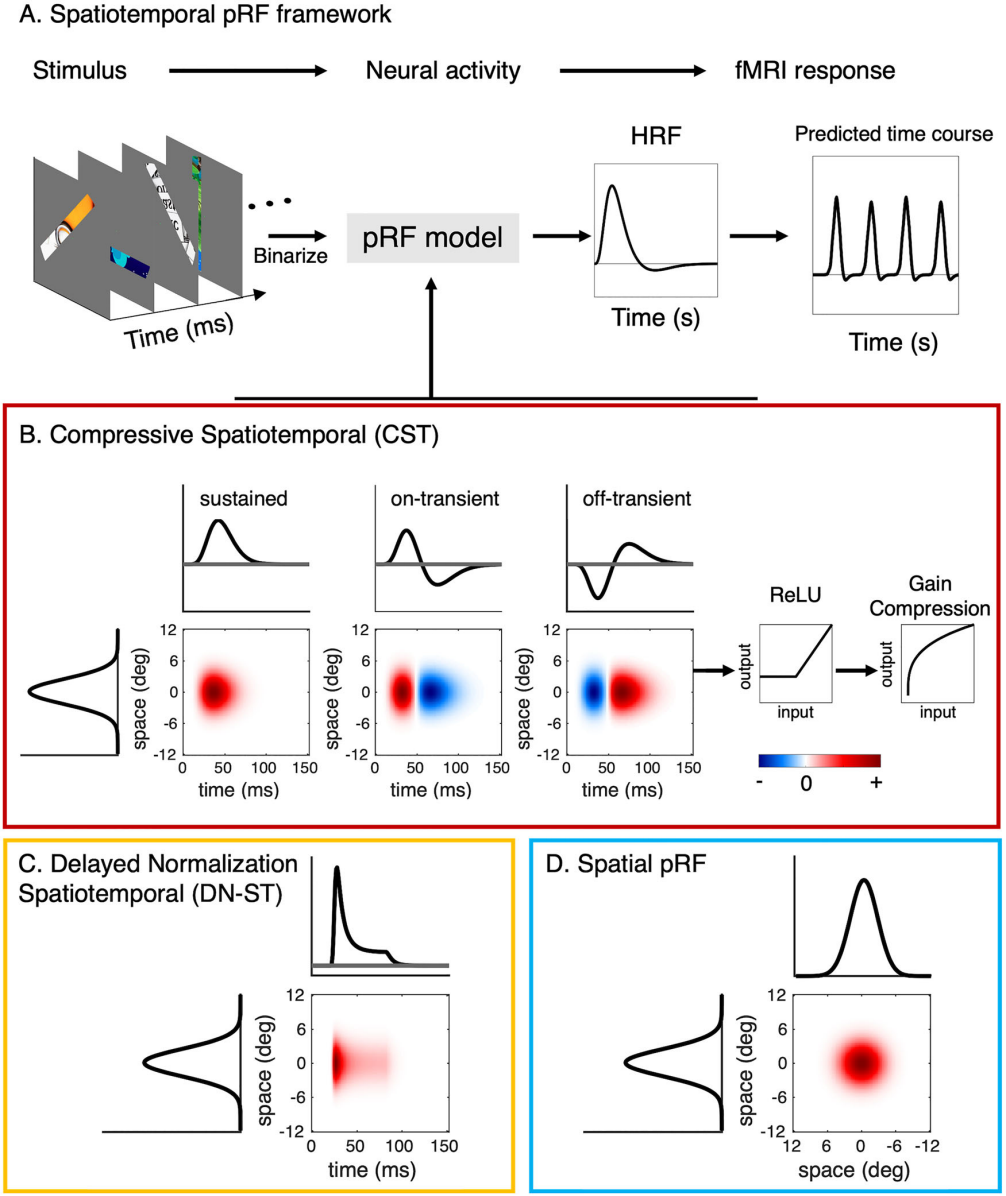
Modeling framework for spatiotemporal population receptive fields. ***A***, Spatiotemporal pRF framework. To predict the fMRI response in each voxel, the binarized visual stimulus is fed into a pRF model to predict the neural population response (temporal resolution of sequence and neural output is discretized into units of 10 ms) and then convolved with the hemodynamic impulse response function (HRF) and resampled to seconds to predict the fMRI response. Unless otherwise stated, the HRF is the voxel-wise optimized HRF (Fig. 2). We implemented and tested three pRF models: ***B***, Compressive spatiotemporal (CST) pRF model. The CST model consists of three spatiotemporal receptive fields that have an identical spatial receptive field (2D Gaussian) and three different temporal receptive field types: sustained (left), on-transient (middle), and off-transient (right). In each channel, the output undergoes rectification (ReLU) removing negative responses and compression by exponentiation. *Red:* positive signal amplitude (a.u.); *Blue:* negative signal amplitude (a.u.). For visualization, only the vertical spatial dimension is shown. ***C***, Delayed normalization spatiotemporal (DN-ST) pRF model. The spatial receptive field is a 2D Gaussian and the temporal receptive field uses a nonlinear impulse response function computed by rectification, exponentiation, and divisive normalization. ***D***, Spatial pRF model is a 2D Gaussian pRF. Here, both spatial dimensions are shown.

First, we predicted neural activities from the stimulus and specific spatiotemporal receptive field model (Fig. 3*A*). Depending on the model, this procedure involved linear and nonlinear computations. Then the predicted neural responses were convolved with an HRF and down sampled to 1 s to predict fMRI responses (Fig. 3*A*). This step is linear and the same HRF was used across all models. Three pRF models were implemented: compressive spatiotemporal (CST, Fig. 3*B*), delayed normalization spatiotemporal (DN-ST, Fig. 3*C*), and spatial (Fig. 3*D*).

This two-step stimulus to neural and neural to BOLD framework is theoretically and implementationally important. From the theoretical perspective, we sought to create a linking model that directly characterizes neuronal responses as well as their spatial and temporal nonlinearities and use it to predict the fMRI response to the stimulus. Implementationally, DN-ST and CST models apply nonlinear operations at the neuronal stage while keeping a linear relationship between the predicted neural activity and BOLD response, as previous studies have shown that the temporal nonlinearities mostly arise from the neuronal activity to the stimulus (Miller et al., 2001; Zhou et al., 2018).

#### Stimulus

The stimulus information is modeled in two spatial dimensions ( XY) and one temporal dimension ( t) and referred to as I(XYt). Each frame of the stimulus sequence was binarized and resized to 61 × 61 pixels and the temporal resolution of stimuli sequences was 10 ms (centisecond). We implemented centisecond rather than a millisecond resolution to reduce computational time.

#### Spatiotemporal population receptive field models

A spatiotemporal pRF is created by taking a pointwise multiplication of the neural spatial and temporal impulse response functions (Adelson and Bergen, 1985; Watson and Ahumada, 1985). To illustrate the spatiotemporal pRF profile in a 2D space, in Figure 3*B,C* we show a cross section of one spatial dimension (*y*-axis) with the temporal dimension (*x*-axis).

We used an identical spatial pRF function for all three models, and only varied the temporal impulse functions specific to each model. The spatial pRF was modeled as a 2D isotropic Gaussian (Dumoulin and Wandell, 2008):

$$\mathrm{spatial}\;\mathrm{RF}(X\;Y)=e^{-({\left(X-x\right)}^{2}+{\left(Y-y\right)}^{2}/2\sigma^{2})}\;$$ (1)

where x and y are the center of the pRF in the visual field, and σ is the spatial extent of the pRF. All units are in degrees of visual angle (°). Figure 3*D* shows an example spatial pRF.

#### Compressive spatiotemporal pRF model

The CST model (Fig. 3*B*) is inspired by neural measurements in macaque V1 (De Valois and Cottaris, 1998; De Valois et al., 2000; Conway and Livingstone, 2003) and human psychophysics (Watson and Robson, 1981; Thompson, 1983; McKee and Taylor, 1984; Hess and Plant, 1985; Watson, 1986). The former showed that different neurons in primate V1 have different temporal receptive fields, which can be characterized by monophasic (sustained) and biphasic (transient) neural impulse response functions (De Valois and Cottaris, 1998; De Valois et al., 2000; Conway and Livingstone, 2003). The latter showed that human temporal sensitivity to visual stimuli can be characterized with a linear system approach with two channels a sustained channel (modeled by a gamma function) and a transient channel (modeled by a difference of gamma functions). As both the onset and offset of visual stimuli are thought to increase neural responses (Horiguchi et al., 2009), we separately modeled onset and offset transient temporal neural impulse response functions. The CST model thus consists of three spatiotemporal channels, each with an identical 2D Gaussian spatial receptive field (spatial RF), and each channel has a different neural temporal impulse response function: sustained, on-transient, and off-transient. The sustained impulse response function models the ongoing neural responses, while the transient impulse response functions computes changes in the neural response and highlights visual transitions. “On” and “Off” responses of the transient impulse response functions were separately modeled to account for increased neural responses with both stimulus onsets and offsets. These three spatiotemporal channels were designed to capture both prolonged and abrupt changes in neural responses to stimuli at specific locations and sizes.

The CST model has three spatial parameters (xyσ), a temporal parameter (*τ*), and a compressive exponent (*n*), where σ is the spatial window, and *τ* is the time-to-peak. In some compressive spatial pRF models (e.g., CSS; Kay et al., 2013), pRF size is reported as $\sigma /\sqrt{n}$; however, here we report pRF size as *σ* because the compressive nonlinearity is applied to spatial and temporal dimensions together.

The CST model is implemented as follows: first, the dot product is applied between binarized spatiotemporal visual input I(XYt) and the *spatial RF* (Eq. 2) at each time point, effectively computing the weighted sum of the spatial overlap between stimulus at time (*t*) and the spatial RF. Then, to predict spatiotemporal neural activity, we convolved the output with three different temporal impulse response functions $h_{i}(t)$, with *i* taking the values 1, 2, and 3. These temporal impulse response functions correspond to sustained $h_{1}(t)$, on-transient $h_{2}(t)$, and off-transient, $h_{3}(t)$ channels. Thus, the predicted spatiotemporal neural activity $r_{i}(t)$ for each of the channels can be expressed:

$$\begin{array}{rl} & r_{i}(t)=h_{i}(t)\times [I(X\;\;Y\;\;t)\cdot \mathrm{spatial}\;\mathrm{RF}(X\;\;Y)]\\ & \;\;\;\mathrm{where}\;i=1\;2\;\mathrm{or}\;3. \end{array}$$ (2)

The sustained neural temporal impulse response was modeled by a gamma function:

$$\;h(t)=\frac{{\left(t/\kappa \tau \right)}^{\left(m-1\right)}e^{-(t/\kappa \tau )}}{\kappa \tau (m-1)!}\;$$ (3)

where t is in milliseconds and τ is a fitted time constant. The values of κ and m parameters were same as previous studies (Watson, 1986; Stigliani et al., 2017, 2019) and were κ=1 and m=9.

The on-transient impulse response function $h_{2}(t)$ was modeled as a difference between two-gamma functions, which yielded a biphasic response. The excitatory component was the same as $h_{1}(t)$ and the inhibitory component was a gamma function with parameters: κ=1.33 and m=10. As such, the peak of the sustained impulse response function is always later than the transient impulse response function. The off-transient impulse response function $h_{3}(t)$ is identical to $h_{2}(t)$ but with the opposite sign.

After spatiotemporal linear filtering, the CST model implements two nonlinearities in each channel: (1) a rectified linear unit (ReLU) and (2) a compressive power-law exponentiation. The purpose of the ReLU is to rectify the negative component of the transient responses (the sustained response is always positive). We reasoned that both on- and off-transient responses will increase neural firing rate and consequently increase BOLD signals. Nonlinear summation in the visual system tends to be subadditive across spatial and temporal domains, as empirical data show that the sum of responses to multiple stimuli over space is lower than the sum of responses to individual stimuli (Kay et al., 2013), and the response to prolonged stimulation is less than the response to multiple individual stimulation with the same overall duration (Zhou et al., 2018, 2019). The predicted neural activity of each channel after nonlinear computations can be expressed as

$$p_{i}(t)=[\mathrm{ReLU}(r_{i}(t)){]}^{n}\;\;\mathrm{where}\;i=1\;\;2\;\;\mathrm{or}\;3;\;\;0.1\leq n\leq 1.$$ (4)

To predict fMRI responses resulting from sustained and transient neural responses, we convolved the predicted neural time courses $p_{i}(t)$ with the HRF and down sampled to 1 s resolution to match the temporal resolution of fMRI measurements. The predicted BOLD response is the weighted sum of the sustained and transient responses:

$$\mathrm{BOL}D_{\mathrm{CST}}=\;\beta_{\mathrm{sus}}(p_{1}(t)*\mathrm{HRF})\;+\;\beta_{\mathrm{tran}}([p_{2}(t)+p_{3}(t)]*\mathrm{HRF}).$$ (5)

From fMRI data and using a GLM approach, sustained ( $\beta_{\mathrm{sus}}$) and transient $\left(\beta_{\mathrm{tran}}\right)$ scaling factors were estimated in each voxel (see also *Model fitting and parameter optimization*). We combined the neural responses of the on- and off-transient channels into a single channel to model the overall transient response profile and estimate one scaling parameter to fit to the BOLD signal. This implementation is specific to the current experiment, and one may separately estimate contributions from on ( $\beta_{\mathrm{Onset}\_\mathrm{tran}}$) and off ( $\beta_{\mathrm{Offset}\_\mathrm{tran}}\;$) transient channels. It is also possible to implement a variant of the CST model with a single β coefficient estimating the gain for the summed neural response of the sustained and transient channels before convolving with the HRF. The CST solver on synthetic data (300 voxels with noise) is similar to its performance on a model with two channels (one for the sustained and one for the transient channels).

#### Delayed normalization spatiotemporal pRF model

The spatiotemporal delayed normalization pRF model (DN-ST) has a 2D Gaussian spatial receptive field like the other models and a temporal impulse function that uses divisive normalization as well as an exponential decay function to model nonlinear neural temporal responses (Zhou et al., 2018, 2019; Groen et al., 2022; Fig. 3*C*).

The DN-ST model has three spatial parameters (xyσ) and four temporal parameters ( $\tau_{1}$, $\tau_{2}$, $n_{\mathrm{DN}}$, and $\sigma_{\mathrm{DN}}$)_._ Here we report pRF size as σ.

Predicted neural activity of the DN-ST model is expressed as

$$p(t)=\frac{{\left|r(t\right)|}^{n_{\mathrm{DN}}}}{{\sigma_{\mathrm{DN}}^{n_{\mathrm{DN}}}+[|r(t)|\times h_{2}(t)]}^{n_{\mathrm{DN}}}}\;$$ (6)

where $\sigma_{\mathrm{DN}}$ is a semisaturation constant and $n_{\mathrm{DN}}$ is an exponent. r(t) is the linear component of the neural response computed as the convolution between the neural temporal impulse response function $\;h_{1}(t)$ and the dot production of the stimulus I(XYt) with the spatial pRF (same as Eq. 2).

$$\;r(t)=\;h_{1}(t)*[I(X\;Y\;t)\cdot \mathrm{spatial}\;\mathrm{RF}(X\;Y)].$$ (7)

Following Zhou et al. (2019), the temporal impulse response function $h_{1}(t)$ was created using a gamma function with a time constant parameter $\tau_{1}$. We implemented the monophasic version of the gamma function to reduce the number of free parameters. We also tested another variant of the DN-ST pRF model with a biphasic neural impulse response function (Zhou et al., 2019; Groen et al., 2022), but this did not reduce the estimation error during simulation (data not shown).

$$\;h_{1}(t)=te^{-t/\tau_{1}}.$$ (8)

Another term in the denominator $h_{2}(t)$ reflects the decay component and is implemented with a low-pass filter that uses an exponential decay function with a parameter $\tau_{2}$:

$$h_{2}(t)=e^{-t/\tau_{2}}.$$ (9)

Finally, the predicted neural activity p(t) was convolved with the HRF to predict fMRI signals:

$$\mathrm{BOL}D_{\mathrm{DN}\text{-}\mathrm{ST}}=\;\beta (p(t)*\mathrm{HRF}).$$ (10)

The β-weights approximate the magnitude of fMRI responses.

#### Spatial pRF model

The linear spatial pRF model is the same as Dumoulin and Wandell (2008) (Fig. 3*D*). It implements the spatial pRF described in Equation (1) to predict the neural response by computing the dot product between the stimulus and the spatial pRF. Then, the neural response is convolved with the HRF to predict the BOLD response. The predicted BOLD response is

$$\mathrm{BOL}D_{\mathrm{spatial}}=\beta ([I(X\;Y\;t)\cdot \mathrm{spatial}\;\mathrm{RF}(X\;Y)]*\mathrm{HRF}).$$ (11)

The Spatial model has three spatial parameters (xyσ).

#### Model fitting and parameter optimization

The pRF parameters of each model at each voxel are determined by a two-stage coarse-to-fine approach that minimize differences between the predicted and measured fMRI time course. The first stage was a grid search procedure where we approximated the spatial RF parameters (*x*, *y*, and *σ*) for each voxel using the default Vistasoft HRF as in Dumoulin and Wandell (2008). The grid search procedure involved enumerating over combinations of potential spatial RF locations and sizes, where *x* and *y* were of a range that covered twice the size of the stimulus: $0^{\circ }\leq x\;y\leq \;24^{\circ }$, 0.4° steps, and *σ* was sampled up to the radius of the stimulus: $0.1^{\circ }\leq \sigma \leq \;12^{\circ }$, log-linear sampled, 96 steps. For the CST model, the grid search also included a range of compressive exponents: *n *= (0.25, 0.5, 0.75, and 1). Additionally, for the DN-ST and the CST models, we used a fixed set of default temporal parameters during the grid search: CST: τ=4.93; DN-ST: $\tau_{1}=0.05\;\;\;\tau_{2}=0.1\;\;\;n_{\mathrm{DN}}=2\;\;\;\sigma_{\mathrm{DN}}=0.1$ same as previous studies (Stigliani et al., 2019; Zhou et al., 2019).

In the second stage, a fine search was performed using a Bayesian adaptive direct search (BADS) algorithm (Acerbi and Ma, 2017). To avoid local minima, we generated three sets of initial parameters using the estimated spatial parameters (xyσ) from the grid search while randomly varying the other parameters. Here, we fit all spatiotemporal parameters simultaneously. Lower and upper search bound of spatial parameters (xyσ) were set to ±5° from the grid search estimation. The search range for remaining parameters were: DN-ST model: $\tau_{1}:[0.01\;\;\;1]\;\;\;\tau_{2}:[0.01\;\;\;1]\;\;\;\sigma :[0.01\;\;\;0.5]\;\;\;n:[1\;\;\;6]$; CST model: τ:[4100], n:[0.11]. Note that for the CST model, temporal ( τ) and compression parameters ( n) were identical across sustained and transient channels. For the fine search, different analyses used the default or optimized HRFs, and a GPU was utilized to accelerate computation time.

After performing the BADS optimization for each set, the parameter set that best explained the fMRI data was selected based on the highest variance explained (*R*^2^).

$$\begin{array}{r} \;R^{2}=1-\frac{\sum_{t=1}^{N}\;{\left(\mathrm{MODEL}(t)-\mathrm{DATA}(t)\right)}^{2}}{\sum_{t=1}^{N}\;\mathrm{DATA}{\left(t\right)}^{2}};\\ \;N:\mathrm{number}\;\mathrm{of}\;\mathrm{timepoints}. \end{array}$$ (12)

### Simulation software

We developed a simulation software to evaluate and ensure the computational validity of spatiotemporal pRF models. The simulation software was built to be compatible with previous validation software for fMRI BOLD responses (Lerma-Usabiaga et al., 2020). The simulation software has two components: (1) Synthesizer**:** generates synthetic fMRI time courses based on the stimulus and a specific pRF model. (2) Solver**:** Given a fMRI time course, a pRF model, and a stimulus sequence, the solver uses an optimization procedure to solve the pRF model parameters that best predict this time course.

**Figure 4.**
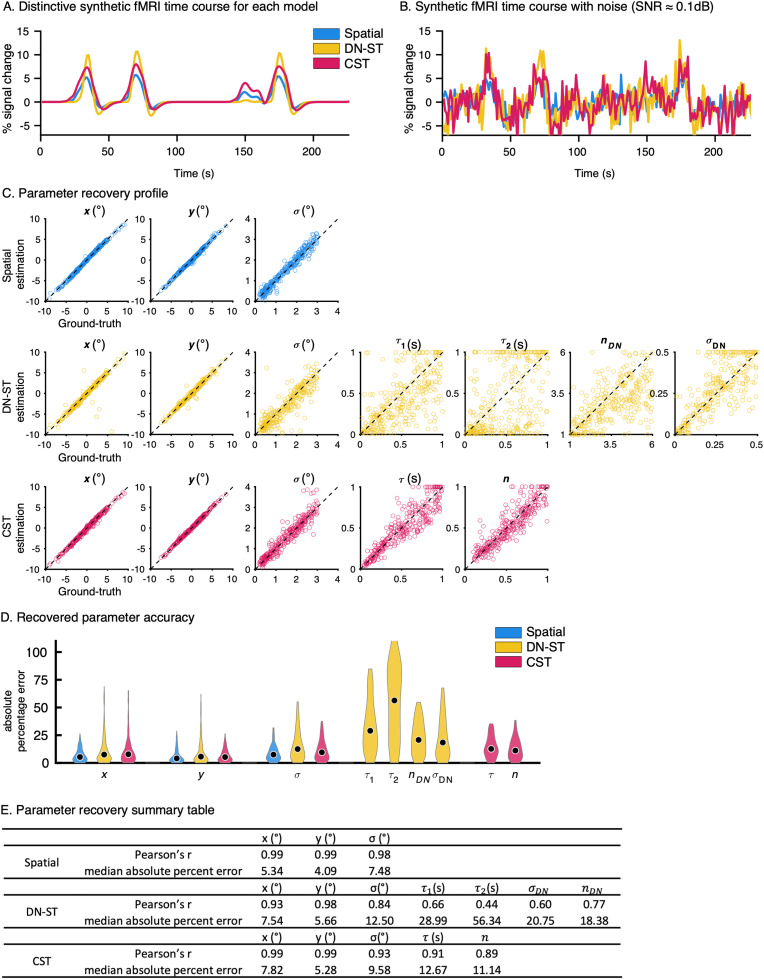
Simulator: Validating spatiotemporal pRF models. ***A,B***, Synthesizer. ***A***, An example time course of noiseless synthetic fMRI time courses generated with the same spatial parameters and experimental paradigm of one run for three different pRF model types (*blue:* Spatial, *yellow:* DN-ST model, *red:* CST model). In each run, there are four bar sweeps across the visual field. ***B***, Simulated time courses from ***A***, with added noise to simulate typical fMRI data. The amount of noise applied to each synthetic time course was matched across three models to yield mean SNR of 0.1 dB. ***C,D***, Solver results. ***C***, Ground-truth parameter versus estimated model parameters for 300 randomly generated pRFs for each model type. Each dot indicates a parameter estimate for a pRF estimated from a noised-added synthesized fMRI time course. Different model types have different numbers of parameters. *Spatial model*: three parameters (xyσ). *DN-ST model:* seven parameters ( $x\;y\;\sigma \;\tau_{1}\;\tau_{2}\;\sigma_{\mathrm{DN}}\;n_{\mathrm{DN}}$), and *CST model:* five parameters (xyστn). The spatial parameters are in units of visual angle (°) and the temporal of $\tau \;\;\tau_{1}\;\tau_{2}$ are reported in units of seconds (s). *Dashed line:* parameter estimate equals ground-truth. ***D***, Violin plots of the accuracy of each of the estimated parameters (absolute percentage error) for 300 simulated pRFs. *Black dots:* median value. For each parameter, outliers beyond 90th percentile were excluded. ***E***, A table summarizing the accuracy of the recovered parameters compared with ground-truth parameters for each of the 300 pRFs for each model. The median absolute percent errors reflect the black dots in ***D***.

**Figure 5.**
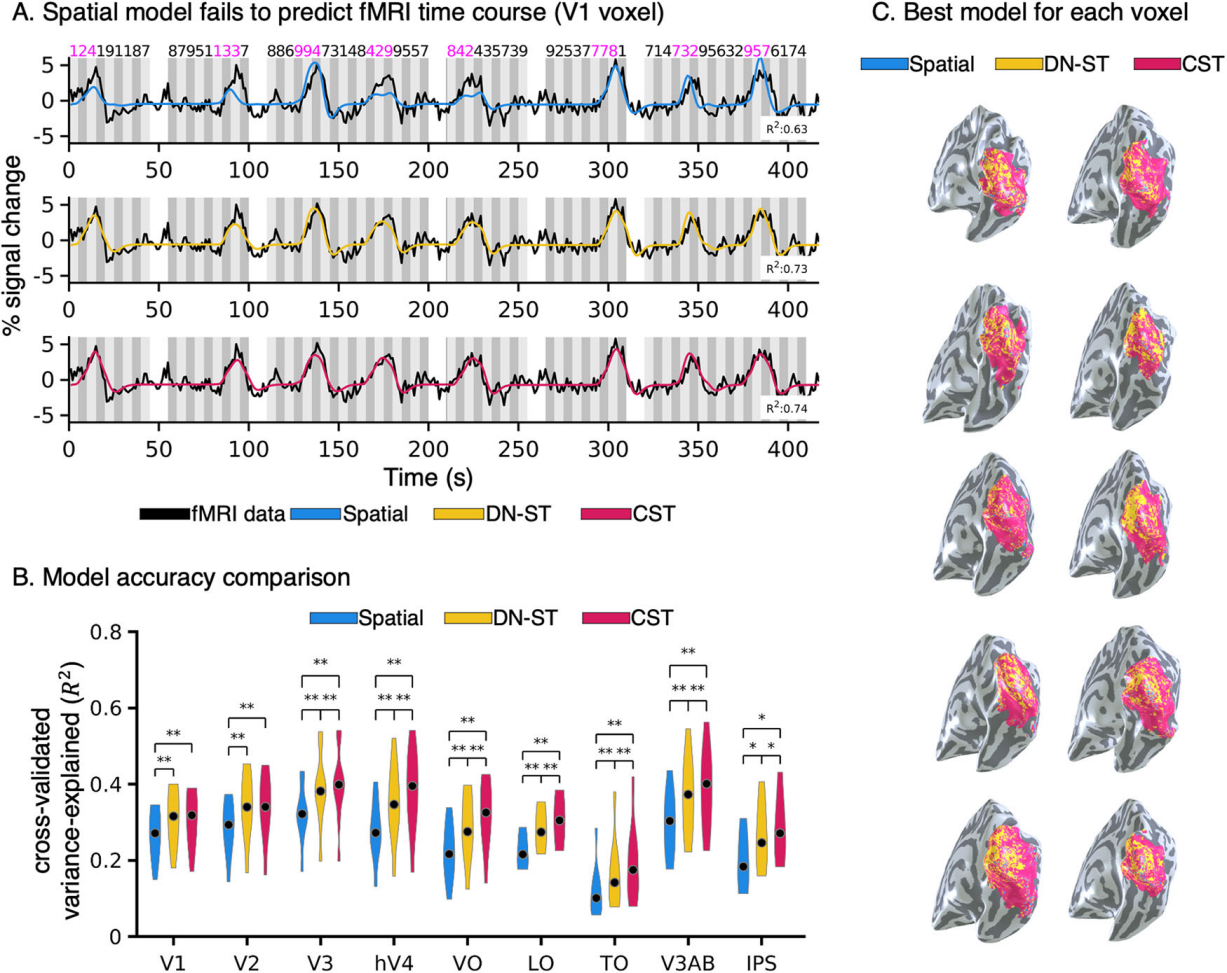
fMRI data: Comparing spatiotemporal pRF models to fMRI voxel data. ***A***, *Black:* An fMRI time course from an example V1 voxel for a segment of the experiment containing two concatenated runs. In each run, there are four bar sweeps across the visual field. The same fMRI data are shown in all rows. *Colored lines:* model predictions; *blue*: Spatial, *yellow:* DN-ST, *red:* CST. *Shaded area:* time points when stimuli were presented. *Numbers at the top:* temporal conditions. *Magenta:* temporal conditions that contribute to each peak. ***B***, Model accuracy comparison. Violin plots of average cross-validated variance explained (*R*^2^) across each participant's voxels and area for each of the three models in nine retinotopic visual areas spanning the ventral, lateral, and dorsal processing streams. *Black dots:* median value. *Asterisk:* Significantly different than spatial model*,* permutation test, *p < 0.05, **p < 0.01. ***C***, Visualization of the best performing model (*R*^2^) for each voxel. Data are shown for each of the 10 participants on their inflated right hemisphere. *Blue:* best model is Spatial, *yellow:* best model is DN-ST, *red*: best model is CST.

To test the robustness of each of the spatiotemporal pRF models, we generated 300 different synthetic fMRI time courses for each model by randomly sampling a wide range of spatiotemporal pRF parameters. All three models shared identical ground-truth spatial parameters. Spatial receptive fields’ spatial parameters of x and y were randomly sampled from a normal distribution spanning an eccentricity range of 0–10° and σ range of 0.2–3° to match the stimulus aperture of our experiment. Additional parameters for the DN-ST and the CST models were randomly sampled from a uniform distribution with the same bounds used in the search algorithm described above. We used the default Vistasoft HRF for the simulation. After generating simulated time courses for each model and parameters, three types of additive noise (white noise, physiological, and low-frequency drift noise) that are commonly found in fMRI signal were applied (Erhardt et al., 2012; Welvaert and Rosseel, 2014; Liu, 2016; Lerma-Usabiaga et al., 2020). The noise magnitude was systematically adjusted to match the range of signal-to-noise ratio (SNR) of ≍0.1 dB (Fig. 4*B*). This range of SNR resulted in a variance explained (*R*^2^) value of approximately 0.3, which was a typical level of *R*^2^ in the empirical spatiotemporal fMRI experiment (Fig. 5*B*).

After generating synthetic fMRI time courses with noise, we tested whether each model can accurately recover its ground-truth spatiotemporal pRF parameters from the synthetized time courses. The parameters were solved using the two-staged coarse-to-fine approach as described above. The identical Vistasoft HRF that was used to generate the synthetic time courses was also used for solving. The performance of each spatiotemporal pRF model was evaluated by comparing the absolute percentage error between the solution and the ground-truth parameters. The absolute percentage error was calculated by taking the absolute difference between the ground-truth and estimated parameter value, and then dividing by the ground-truth value. We also used Pearson's correlation coefficient (*r*) to compare parameter estimates of each model and the ground-truth parameter values.

### Estimating spatiotemporal pRFs across voxels of a visual area and across cortex

As different voxels' pRFs are centered in different locations in the visual field, to examine the aggregate properties of spatiotemporal receptive fields across voxels of a visual area, we zero-centered all pRFs spatial locations to x=0 and y=0. This allowed us to examine the distribution of spatiotemporal pRFs' spatial and temporal windows in a visual area irrespective of their location in the visual field. These zero-centered spatiotemporal pRFs were then averaged across all voxels within each visual area in each participant and then across participants. For the CST model, this analysis was performed separately for the transient and sustained channels. Note that both sustained and transient impulse response functions share the same temporal parameter τ. As the on- and off-transient spatiotemporal are identical except for their sign, for simplicity we only plotted the on-transient channels.

To assess the relationship between spatial and temporal windows of spatiotemporal pRFs, we measured each voxel's temporal processing window size by calculating the FWHM of the sustained neural temporal impulse function. Then, we generated cortical maps of the pRF size and temporal window estimates. The map was generated for each participant and then transformed to FreeSurfer's average cortical surface using cortex-based alignment (Fischl et al., 1999) and averaged across the 10 participants to generate the group average cortical map.

#### Statistical testing

To compare model performance, we used a threefold-cross-validation approach and averaged *R*^2^ across folds. In each fold, two-third of the data (six runs concatenated) was used to estimate model parameters, and the left-out one-third of the data (three runs concatenated) was used to estimate the variance explained by the model. The statistical significance of differences in model performance was computed by applying a Fisher's permutation test with 50,000 iterations and correcting the *p*-values for false discovery rate. To keep the same number of voxels for each model, if the analysis required comparison across different models, we did not exclude any of the fMRI voxels based on the variance explained. When examining the results of a particular model, we excluded voxels that account for less than 10% of the variance explained by that model. When reporting empirical fMRI results of the temporal parameter (τ) of the CST model, voxels with ill-posed estimates that are constrained at the boundary (*τ* = 100, centisecond) were excluded.

To test if pRF size and temporal window significantly (if *p* < 0.05) vary across visual areas, we computed the median pRF size and temporal window for each visual area, for each subject. Using these median values, we performed linear mixed model (LMM) analyses with participants as a random effect and a fixed slope (i.e., the intercept but not the slope can vary across participants): *temporal window ∼ visual area + *(1 *| participant*) and *pRF size ∼ visual area + *(1 *| participant*).

#### Analysis of pRF temporal window across eccentricities in early visual cortex

To quantitatively evaluate the relationship between temporal window and eccentricity in V1, V2, and V3 (Fig. 10*B*), we binned pRFs into eccentricity bins of 2° (visual angle) for each participant. As a result, different numbers of voxels contribute to each datapoint, depending on the number of voxels for each participant and eccentricity bin. Then, we used an LMM with participant as a random effect: *temporal window ∼ eccentricity + *(1 *| participant*). This LMM fits a single slope across all participants and allows each participant to have a distinct intercept. Note that this is different from how we report median pRF temporal windows in Figure 8*B*, where we calculated median of pRF windows across all eccentricities on the unbinned data. These differences in analyses may contribute to quantitative differences in temporal window estimates between Figures 8*B* and 10*B*.

#### Software accessibility

The analysis code (https://github.com/VPNL/stPRF) and the pRF simulation software (https://github.com/vistalab/PRFmodel) are all accessible online.

#### Data accessibility

The data and additional information are accessible on the Open science framework (https://osf.io/3gwhz/).

## Results

### Validating spatiotemporal pRF models through simulation

To evaluate the robustness and validity of the spatiotemporal pRF modeling framework, we developed a simulation software with two purposes. First, to simulate experimental paradigms and test if they can be used to differentiate between predictions of different pRF models. Second, to validate the accuracy of the framework by testing if it recovers ground-truth pRF model parameters from simulated fMRI time courses with noise. By understanding how well our framework can recover parameters in simulated scenarios, we can define the scope of model interpretability given our experimental design and number of measurements.

### Synthesizer: different pRF models predict distinctive time courses to identical visual stimuli

Using the stimulus design in the spatiotemporal pRF mapping experiment (Fig. 1), 300 noiseless and 300 noise-added synthetic fMRI time courses were generated for each of the pRF models: spatiotemporal pRF models (DN-ST and CST) and the conventional spatial pRF model (Dumoulin and Wandell, 2008). To compare how the temporal parameters affected fMRI time courses, synthetic fMRI time courses for each model used the same spatial parameters ( xyσ) while varying model-specific parameters for DN-ST and CST.

We found that even with the same stimuli and identical spatial parameters, distinct synthetic fMRI time courses were generated for each model. An example run (out of nine runs in total) of three noiseless synthetic time courses, one for each of the different pRF models with the same spatial parameters, is shown in Figure 4*A*. All the example time courses had four distinct peaks at similar times during the experiment, corresponding to the four times the bar swept across the pRF. However, the different models generated distinctive fMRI time courses in terms of their amplitudes, latencies, and widths and these differences were preserved when typical fMRI noise was applied (Fig. 4*B*). The different synthetic time courses confirmed that different types of pRF models can generate fMRI time courses that can be experimentally differentiated with our stimulus design.

### Solver: testing the accuracy and robustness of the spatiotemporal pRF framework vis-à-vis ground-truth

We next tested whether the solver accurately recovers ground-truth pRF parameters from synthetic fMRI time courses. Specifically, using a synthetic fMRI time course with additive noise and a stimulus sequence as model inputs, the solver estimates the specific model parameters for each pRF. To evaluate the solver's accuracy, we compared the estimated parameters to the ground-truth parameters that generated the synthetic time courses. Both the synthesizer and the solver used the same pRF model.

For noiseless synthetic fMRI time courses such as the ones in Figure 4*A*, the solver successfully recovered the spatiotemporal pRF parameters of all models with more than 99% accuracy. This result validates the analysis code and indicates that the solver can resolve pRF parameters from the planned experimental sequence for all model types.

When a typical level of empirical fMRI noise was added to the synthetic fMRI time courses, the models are still able to successfully recover the pRF parameters. The solver accurately recovered the spatial parameters ( xyσ, Fig. 4*C*) of all models. Significant correlations were found between the predicted and ground-truth spatial parameters (Fig. 4*E*, all correlations are significant *p* < 0.0001) for all three models. Estimated pRF center positions (xy) were highly accurate (Fig. 4*C*, estimated vs ground-truth along the dashed equality line). The median absolute percentage error (MAPE) for pRF centers (xy) was 4.09–7.82% across the models (Fig. 4*D,E*). PRF size ( σ) was also accurately recovered for all models (Fig. 4*C–E*), yet with a higher MAPE of 7.48–12.5%. The higher accuracy of xy than σ estimates is consistent with previous reports of lower accuracy in estimating pRF size than pRF center positions (Lage-Castellanos et al., 2020; Lerma-Usabiaga et al., 2020). Additionally, we tested whether the recovered spatiotemporal parameters interact with each other and may bias estimates of individual parameters. There were no significant relationships between estimates of CST model parameters ( στn, *p* > 0.5) suggesting that CST parameter estimates are unbiased. However, for the DN-ST model, we observed a significant negative coupling between $\sigma_{\mathrm{DN}}$ and $\tau_{1}$ (*F*_(1,298)_ = 5.57, *p* < 0.05), and $\sigma_{\mathrm{DN}}$ and $\tau_{2}$ (*F*_(1,298)_ = 12.09, *p* < 0.001). This suggests that given the current experimental design, the solutions of the temporal parameters of the DN-ST are not independent from one another.

Although the solver was overall less accurate for estimating the temporal parameters than spatial parameters from noisy synthetic data, it successfully recovered the temporal ( τ) and compression ( n) parameters from the CST model, and the temporal parameters for the DN-ST model ( $\tau_{1}\;\tau_{2}\;\;\sigma_{\mathrm{DN}}\;\;n_{\mathrm{DN}})$, with better estimates for the CST than the DN-ST model (Fig. 4*C,D*; all correlations between estimated and ground-truth parameters are significant *p* < 0.0001). Remarkably, the CST model was able to resolve the temporal receptive field parameter ( τ) with an MAPE < 13% (Fig. 4*D*,*E*). Despite the sluggish nature of hemodynamic responses, the stimulated result suggests that for ∼200 ms temporal receptive fields, there is only a ±26 ms margin of error. The compression parameter, n, was also reliably estimated. The estimates of the DN-ST model temporal parameters were also significantly correlated with the ground-truth temporal parameters (Fig. 4*C*,*E*, *p* < 0.0001), but the estimates were less accurate than the CST model, with $\tau_{2}$ being the least accurate parameter (Figs. 4*D*, 1*E*). This variability of $\tau_{2}$ estimates may be due to an insufficient number of conditions with prolonged presentation, as the $\tau_{2}$ parameter mainly controls the temporal decay rate of the response to prolonged stimulus durations.

To assess the impact of the experimental design choice on parameter estimation, we also tested how well parameters are recovered from a different pRF mapping experiment that uses an image presentation rate of 8 Hz (Toonotopy; Finzi et al., 2021). For the same models and pRFs, we generated simulated time courses with noise for the Toonotopy experiment (data not shown). Results from the Toonotopy experiment indicated that pRF locations were similarly well-estimated for all models (MAPE for x and y were less than 12%). However, MAPE for pRF size ( σ) estimates from the Toonotopy experiment were 48.23% for the DN-ST and 42.52% for the CST. Additionally, the estimation errors for the remaining parameters exceeded 35% for the DN-ST model and exceeded 30% for the CST model. This suggests that Spatiotemporal mapping experiment (Fig. 1) is more suitable for estimating spatiotemporal parameters than the Toonotopy experiment.

### Spatiotemporal pRF models outperforms spatial only pRF model

We next assessed how well the three pRF models: Spatial, DN-ST, and CST, predicted the empirical fMRI time courses in the Spatiotemporal mapping experiment (Fig. 1) in each of our 10 participants. Note that the time courses predicted from the Spatial model depend only on the location and duration of the stimulus, whereas the predictions of the DN-ST and CST models also depend on the spatiotemporal aspects of the stimulus at each location, resulting in more complex fMRI responses.

The spatiotemporal stimulus design evoked a wide range of BOLD time courses in voxels of the visual system. Based on each voxel's fMRI time course, pRF parameters for each of the three models were estimated. Results show that fMRI responses at the voxel level were well captured by the DN-ST (example voxel, Fig. 5*A*, yellow) and CST (example voxel, Fig. 5*A*, red) models. However, the Spatial model failed to predict the fMRI time courses in multiple ways. For example, as shown in the example V1 voxel (Fig. 5*A*, blue), the Spatial pRF model underpredicted responses for stimulus conditions with fast temporal rates (temporal conditions 1, 2, and 4), replicating previous findings by Stigliani et al. (2017), and overpredicted responses for prolonged stimulus conditions (temporal condition 5). In contrast, the DN-ST and CST models were able to predict both the amplitude and width of these peaks that the Spatial model failed to predict.

We quantified how well the various models predicted the experimental time courses in each voxel using a threefold-cross-validation approach. On average, the Spatial, DN-ST, and CST models accounted for 25, 30, and 33% of the variance, respectively. Each model's accuracy across multiple retinotopic visual areas is plotted in Figure 5*B*. The DN-ST and CST models outperformed the Spatial model in predicting fMRI responses in single voxels of all tested visual areas. Indeed, the threefold-cross-validated variance explained (*R*^2^) by the DN-ST and CST models was significantly higher than the Spatial model (Fig. 5*B*; permutation test, V1, V2, V3, hV4, VO, LO, TO, V3AB, *p* < 0.01; IPS, *p* < 0.05). Comparing the spatiotemporal pRF models, the CST model outperformed DN-ST model across all visual regions (permutation test, V3, hV4, VO, LO, TO, V3AB, *p* < 0.01; IPS, *p* < 0.05) except V1 and V2, where performance did not differ significantly between the two spatiotemporal models.

To examine if there were systematic and spatial differences in how well models fit the data across cortex, we numerically compared the variance explained by the three pRF models in each voxel. Then, we visualized on the cortical surface which model best predicted the response of each voxel (Fig. 5*C*). We found that: (1) in all participants, there were almost no voxels for which the Spatial model was the best model (very small number of blue voxels in Fig. 5*C*), (2) for most of the voxels, the CST model was the best (except for voxels around the occipital pole for which the DN-ST model was better), and (3) consistent with the quantitative analyses in Figure 5*B*, the advantage of the CST over DN-ST model was more pronounced in later visual areas. To further assess the robustness of the spatiotemporal pRF models, we compared model performance across the same participants using the Toonotopy fMRI data, which had a shorter temporal duration (2-s per location) and an image presentation rate of 8 Hz. Using the Toonotopy fMRI data (data not shown), we found that both spatiotemporal pRF models significantly outperformed the Spatial model in all visual areas (permutation test, V1, V2, V3, hV4, VO, LO, TO, V3AB, *p* < 0.01; IPS, *p* < 0.05), and the CST model had significantly higher accuracy than both the DN-ST and Spatial models (permutation test, V1, V2, V3, hV4, VO, LO, TO, V3AB, *p* < 0.01; IPS, *p* < 0.05).

Together, these analyses: (1) support our hypothesis that both spatial and temporal aspects of the stimulus contribute to fMRI signals at the voxel level, (2) show that both DN-ST and CST spatiotemporal pRF models can capture spatial and temporal dynamics of neural responses, and (3) suggest that considering the nonlinear temporal dynamics, both at the stimulus and neuronal level in subsecond resolution into a pRF model is crucial for accurately predicting fMRI timeseries at the voxel level.

### Spatial estimates across models

**Figure 6.**
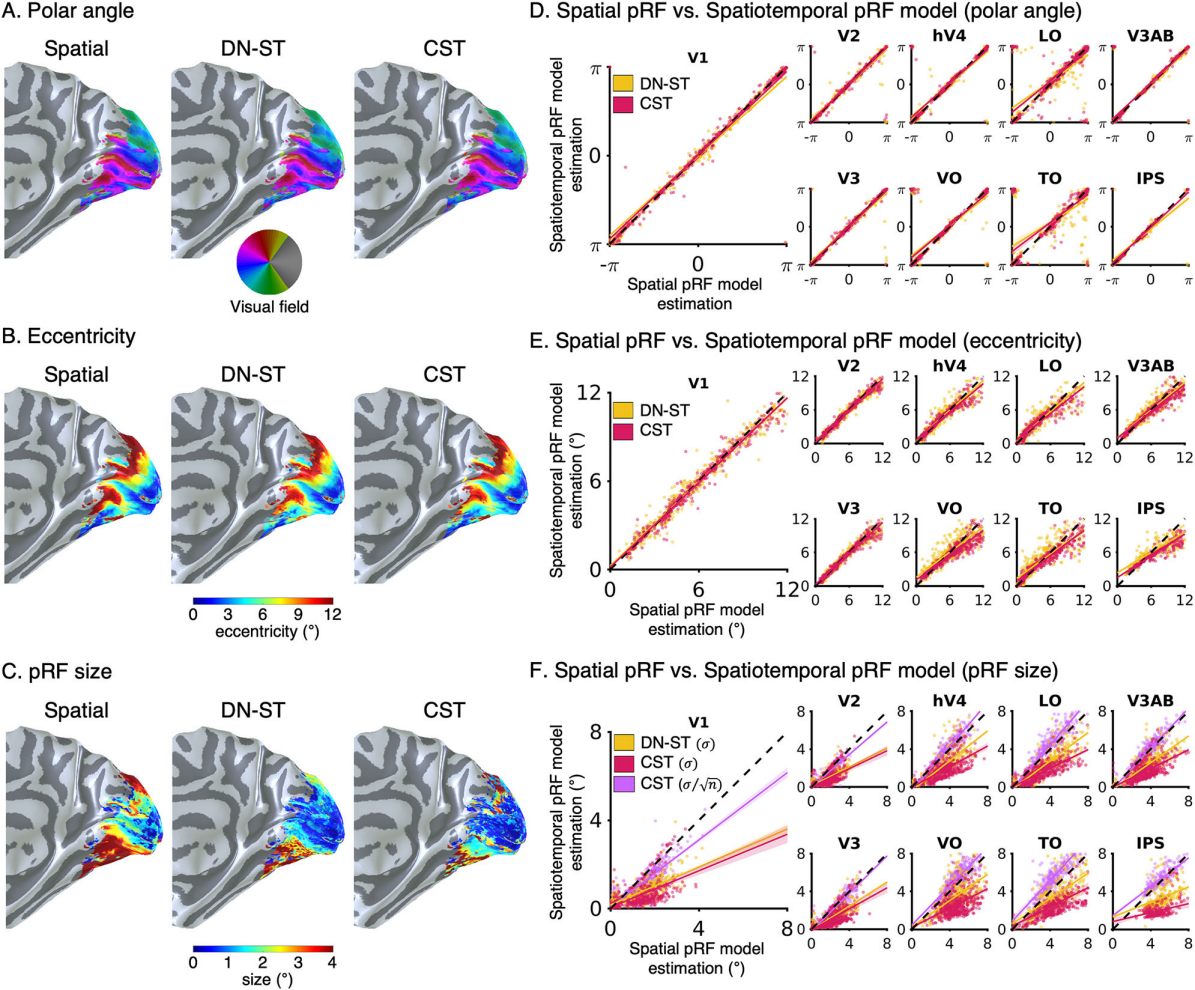
Spatial pRF parameters estimated for the Spatial, DN-ST, and CST models are similar. ***A***, Polar angle, ***B***, eccentricity, and ***C***, pRF size maps on the medial view of the right inflated cortical surface of an example participant for each of the three models. ***D–F***, Scatter plots comparing pRFs from spatiotemporal pRF models in *yellow:* DN-ST and *red:* CST (*y*-axis) to estimates from the spatial pRF model (*x*-axis) for each of nine visual areas. Each dot indicates the parameter estimate for one voxel. *Colored lines:* linear regression fits. For visualization, we randomly sampled 50 voxels from each participant for each visual area. *Dotted black line:* reference line of identical estimates. ***D***, Polar angle. ***E***, Eccentricity. ***F***, pRF size (σ) and adjusted pRF size ( $\sigma /\sqrt{n}$) for the CST model (*purple*).

As spatiotemporal pRF models are newly developed, it is important to test if the spatial pRF parameters they estimate are topographically organized and replicate well-established retinotopic maps in human visual cortex. Thus, we next used the estimated spatial parameters to generate polar angle, eccentricity, and pRF size maps for each participant and model (Fig. 6*A–C*). We also quantitatively compared the estimated pRF position (polar angle), eccentricity, and size in degrees of visual angle (°) for the DN-ST and CST pRF models to those of the Spatial model (Fig. 6*D–F*).

We found that all three models produced robust and consistent estimates of each voxel's pRF positions. From pRF center positions (xy), we generated polar angle and eccentricity maps, which were indistinguishable across the three pRF models and had the expected topography of phase reversals and eccentricity (see example participant, Fig. 6*A,B*). Moreover, all models generated similar topographies of pRF size estimates with increasing sizes along a posterior–anterior axis (Fig. 6*C*). Although in high agreement, pRF size estimates ( σ) from spatiotemporal pRF models were smaller than the Spatial pRF size estimates in all tested visual areas (Fig. 6*F*). In compressive models with a static nonlinearity compression ( n), researchers (Kay et al., 2013) typically report the adjusted pRF size ( $\sigma /\sqrt{n})$. Comparison of the adjusted pRF size ( $\sigma /\sqrt{n})$ from the CST model to the Spatial model yielded similar estimates (Fig. 6*F*, purple). This result suggests that the smaller pRF size estimates of the spatiotemporal pRF models are mainly due to compressive nonlinearities within the models.

Overall, the reliable estimate of spatial pRF properties across models suggests that the incorporation of additional temporal parameters into the pRF model does not reduce the statistical power to map spatial pRFs in human visual cortex.

### Differences in temporal estimates cannot be explained by differences in HRFs

We next characterized the temporal parameters of spatiotemporal pRFs across voxels of the visual system. Under our experimental paradigm, the simulations show that temporal parameters of the CST model were more accurately recovered than the DN-ST model (Fig. 4*C,D*) and experimental data revealed that the cross-validated variance explained of fMRI responses was higher for the CST than DN-ST model for most voxels (Fig. 5*B*). Thus, in the following sections, we report the temporal and spatiotemporal characteristics estimated from the CST model.

**Figure 7.**
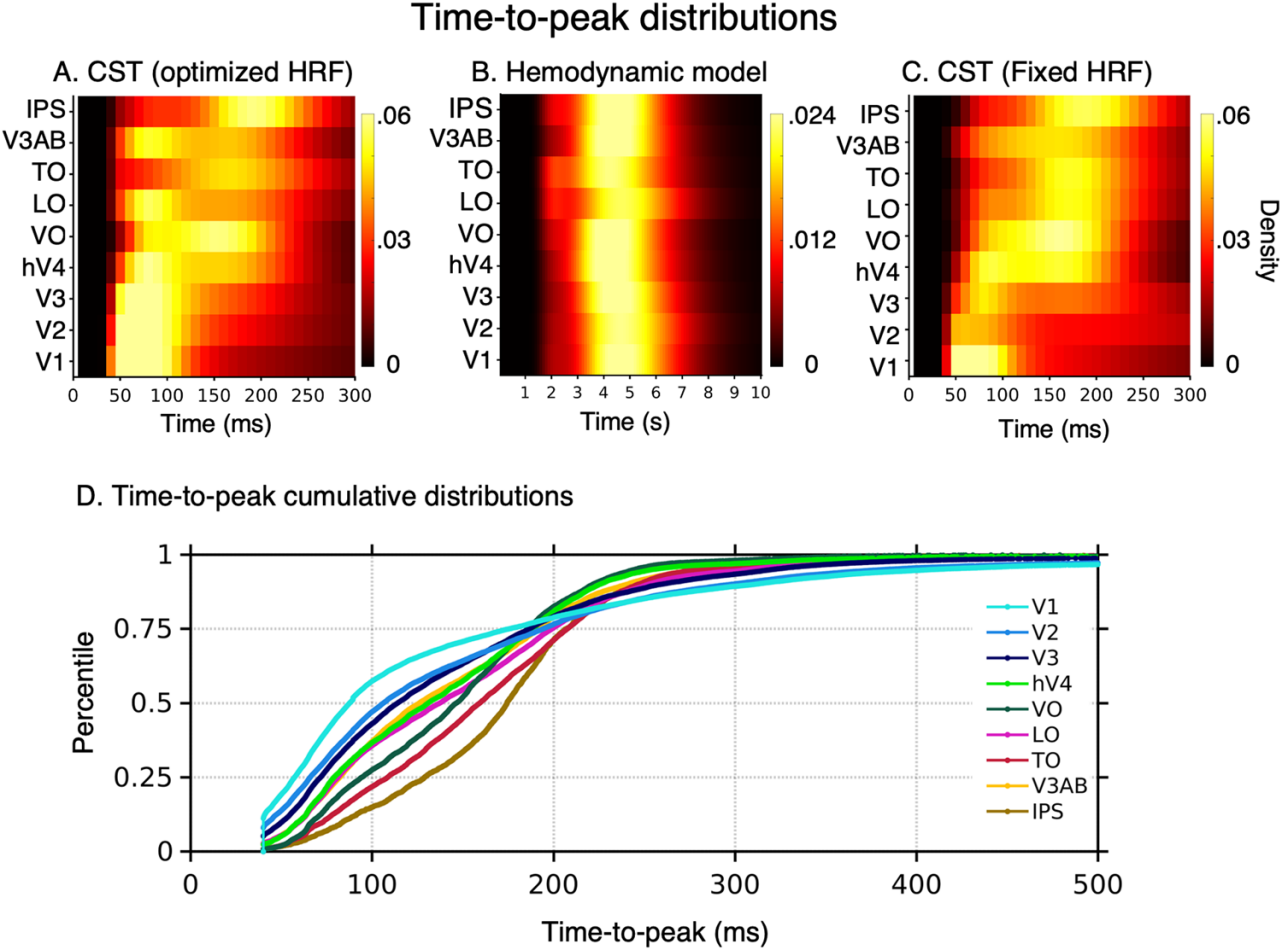
Temporal pRF estimates from the CST model. ***A–C***, Each row shows a voxel-wise time-to-peak distribution for each visual area. The distributions were averaged across participants within each visual area. ***A***, Time-to-peak ( τ) distribution estimated from the CST model, using an optimized HRF for each voxel. ***B***, The distribution of optimized HRF time-to-peak. Note that unlike the CST model, the temporal resolution for the hemodynamic model is in seconds. ***C***, Time-to-peak estimated from the CST model using a single default Vistasoft HRF for all voxels. ***D***, The cumulative time-to-peak distribution across visual areas from the CST model with optimized voxel-wise HRF for each of the nine visual areas. The datapoints from all participants were included for each visual area.

We assessed the time-to-peak from the temporal parameter τ of the CST model and the temporal processing window from the FWHM of the sustained neural temporal impulse function. The neural time-to-peak ( τ) was estimated in each voxel and visual area, then the distribution of neural time-to-peak across voxels of an area averaged across participants for early visual areas (V1, V2, V3) as well as intermediate and later visual areas in the ventral (V4, VO), lateral (LO, TO), and dorsal streams (V3AB, IPS) was plotted. As evident from Figure 7*A*, neural time-to-peak increases from earlier to later areas but also varies within each visual area.

As we estimated for each voxel its optimized HRF (Fig. 2), it is possible that this variability in the time-to-peak is of hemodynamic rather than neural origin. To examine this possibility, we conducted two additional analyses. First, we analyzed the distribution of time-to-peak of the hemodynamic functions within and across areas. We reasoned that if the temporal variability is of hemodynamic origin, then the distributions of optimized HRF time-to-peak will mirror the distributions of the estimated neural time-to-peak. However, this was not the case. The distribution of hemodynamic time-to-peak ranged from 3 to 6 s and was similar across all tested visual areas (Fig. 7*B*). Critically, this distribution (Fig. 7*B*) did not show the between-area differences in neural time-to-peak estimated by the CST model (Fig. 7*A,C*). Second, we repeated the calculations of the CST neural time-to-peak ( τ) using a fixed HRF for all voxels (Vistasoft default HRF). We hypothesized that if the estimated neural time-to-peak interacts with the estimated HRF parameters for each voxel, then using a fixed HRF will qualitatively change the estimates of neural time-to-peak within and across areas. However, we found that using a fixed HRF for all voxels produces quantitative but not qualitative changes in estimates of neural time-to-peak (Fig. 7*C*). Specifically, using a fixed HRF increased the within-area variability of neural time-to-peak estimates (e.g., compare V3 in Fig. 7*C* to 7*A*), but did not change the observation that the time-to-peak progressively increases across visual the hierarchy. These analyses give us confidence that our approach enables estimating neural temporal properties. In the next sections, we will examine in detail the temporal and spatiotemporal parameters from the CST model with the voxel-wise optimized HRF.

### Hierarchical temporal latency delay across visual streams

An open question is how the time-to-peak and processing temporal window changes across the visual hierarchy and processing streams. A feedforward model of the visual system predicts that the time-to-peak will progressively increase across the visual hierarchy (Nowak and Bullier, 1997). Additionally, the ventral stream which is associated with perception of invariant properties of people or objects (such as their identity or category) may be slower and have longer times-to-peak than other streams, like the lateral stream, which is thought to process motion and dynamical aspects of the visual scene (Van Essen and Gallant, 1994; Weiner and Grill-Spector, 2013; Pitcher and Ungerleider, 2021; Wurm and Caramazza, 2022).

We found differences in time-to-peak both across the hierarchy and across streams, thus finding evidence supporting both hypotheses. Across the hierarchy, time-to-peak increased from earlier (V1, V2, V3) to later visual areas in each processing stream generating a cascade of neuronal signal propagation. We found that voxels in V1 had the earliest time-to-peak latencies ( τ∼50–110ms Fig. 7*A*), and that time-to-peak was less than 100 ms for more than 50% of V1 voxels (Fig. 7*D*). V2 followed V1 ( τ∼50–120ms), and V3 followed V2 ( τ∼50–130ms). In the ventral visual stream, later time-to-peak was observed in voxels of intermediate visual area hV4 ( τ∼70–170ms Fig. 7*A,D*, green) which were followed by responses in a late visual area, VO ( τ∼80–200ms, Fig. 7*A,D*, dark green). Similar trends were observed in the lateral (LO to TO, pink and red in Fig. 7*D*) and the dorsal streams (V3AB to IPS, yellow and brown in Fig. 7*D*). Timing of the intermediate areas did not vary across streams with hV4 (ventral), LO (lateral), and V3AB (dorsal) having similar time-to-peaks. However, we found differences across later areas of the different streams. TO (lateral) had a later times-to-peak than VO (ventral), and IPS had the latest time-to-peak ( τ∼160–230ms) compared with other tested visual regions (Fig. 7*A,D*, brown).

### Spatiotemporal processing across visual streams

Crucially, the CST model allows us to examine the spatiotemporal properties of pRFs across visual streams. Prior research has suggested that both spatial receptive field sizes (for review, see Wandell and Winawer, 2015) and temporal windows (Hasson et al., 2008; Honey et al., 2012; Murray et al., 2014; Baldassano et al., 2017) progressively increase across the visual hierarchy. However, before the current framework, it was not possible to estimate spatial and temporal pRF sizes in degrees and milliseconds directly at the voxel level.

**Figure 8.**
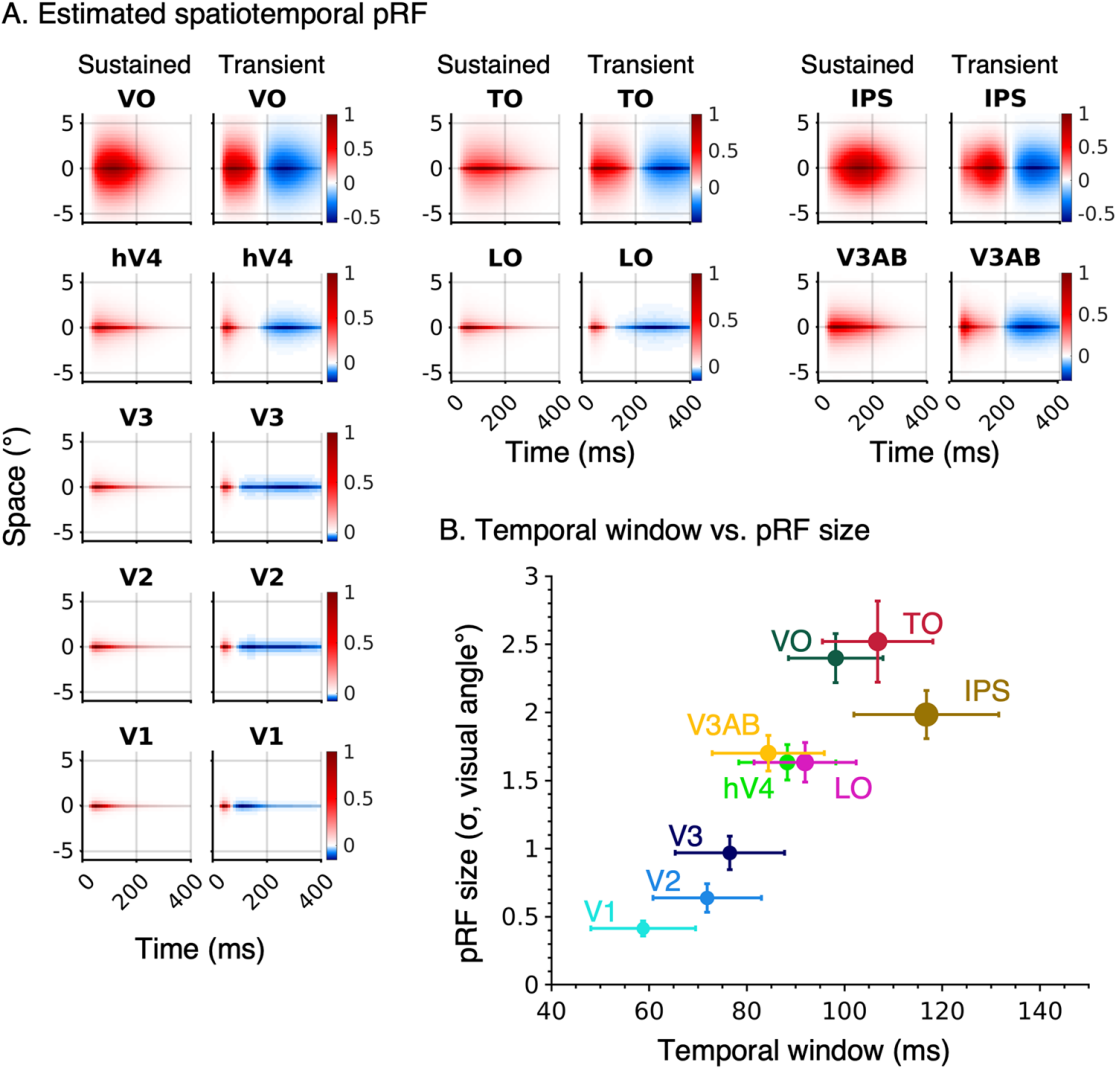
Spatiotemporal pRF characteristics for nine visual areas spanning three visual streams. ***A***, Spatiotemporal pRFs for sustained and on-transient pRFs averaged across voxels and then across participants for each visual area. The spatial location of all pRFs was zero-centered to (0,0) before averaging. The *x*-axis represents time (ms), and the *y*-axis represents a cross section of visual space (°). The red–white–blue colormap is the average of spatiotemporal pRFs, where red and blue represent the positive and negative amplitudes, respectively. ***B***, PRF temporal window (FWHM of the sustained temporal impulse response function) versus pRF size ( σ deg). *Colored dots:* median values for each visual area. Marker size is related to median values of the spatiotemporal compressive exponent (*marker size* ∼ 1/*n*). That is, larger markers indicate larger compression. *Error bars*: ±1 SEM across 10 participants in each dimension.

Figure 8*A* shows the average sustained and transient spatiotemporal pRF across voxels of a visual area (see Materials and Methods, estimating spatiotemporal pRFs across voxels of a region and across cortex). Earlier visual areas had smaller pRF sizes and shorter temporal windows compared to later visual areas for both sustained and transient channels. For example, ascending the ventral stream (V1 → V2 → V3 → hV4 → VO), spatiotemporal pRF windows progressively increased both spatially and temporally (Fig. 8*B*). Differences between visual areas were statistically significant both for pRF size (*F*_(1,84)_ = 57.15, *p* < 0.001) and temporal window (*F*_(1,84)_ = 33.69, *p* < 0.001). In the sustained channel, V1 pRFs integrate visual information spatially across 0.41° ± 0.05° (median ± SEM) and temporally across 58.77 ± 10.71 ms, hV4 across 1.63° ± 0.13° and 88.27 ± 9.93 ms, and VO across 2.39° ± 0.18° and 98.19 ± 9.66 ms (Fig. 8*A,B*). Likewise, in the lateral stream, TO spatiotemporal pRFs were larger in space and time compared to LO, and in the dorsal stream, IPS spatiotemporal pRFs were larger in space and time than V3AB. In the transient channel, we find a similar progression (Fig. 8*A*, right).

To quantitatively compare the spatiotemporal pRF size and temporal window estimates, we computed the average pRF size and average temporal window of each visual area (Fig 8*B*). The comparison showed a positive relationship, revealing a general trend of progressively increasing spatial and temporal windows along the visual hierarchy. Specifically, spatiotemporal pRF size and temporal window progressively increased from V1 to V2 to V3 (Fig. 8*B*, blues). Intermediate areas of all three visual streams (hV4, LO, and V3AB) had similar spatial and temporal pRF sizes which were larger spatially, and longer temporally than V1–V3. The spatiotemporal properties of later visual areas (VO, TO, and IPS) differed across streams. Notably, spatiotemporal pRFs in VO, a ventral area, were smaller spatially and shorter temporally (Fig. 8*B*, dark green) than pRFs in TO, a lateral area (Fig. 8*B*, red), whereas IPS had pRFs that were smaller spatially, but longest temporally (Fig. 8*B*, brown). In addition, the amount of spatiotemporal compression increased along the visual hierarchy (Fig. 8*B*, marker size ∼ 1/*n*). The compressive exponent (*n*) in V1: 0.28 ± 0.03, V2: 0.25 ± 0.06, V3: 0.24 ± 0.06, hV4: 0.22 ± 0.04, VO: 0.21 ± 0.03, LO: 0.19 ± 0.02, TO: 0.18 ± 0.02, V3AB: 0.21 ± 0.02, IPS 0.14 ± 0.02, which is comparable to the increase in spatial compression levels from early to late visual areas (Kay et al., 2013). These results illustrate an interesting coupling between spatial and temporal receptive field windows that progressively increase within each of the three processing streams and diverge across streams in the later visual areas.

**Figure 9.**
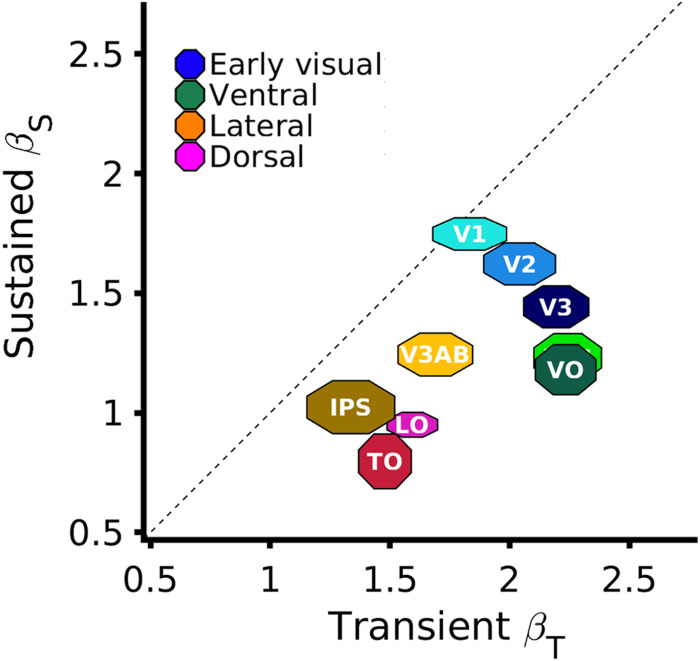
Contributions of sustained and transient responses across the visual hierarchy. Average sustained (*y*-axis) and transient (*x*-axis) response amplitudes (*β* weights) for each visual area estimated by the CST model. Marker size in each dimension indicates Median ±1 SEM across 10 participants. *Dashed line:* equal sustained and transient contributions.

When comparing the contributions of the sustained and transient channels across the visual hierarchy, we found stronger transient responses than sustained responses in all visual areas except for V1 (Fig. 9), which is similar to the findings of Stigliani et al. (2017). We further examined contributions of sustained and transient channels across eccentricities in early visual cortex (data not shown). Consistent with previous findings (Horiguchi et al., 2009; Stigliani et al., 2017), we observed balanced sustained and transient contributions between 3° and 12° of eccentricity in V1, but we found higher transient responses in the central 5° than Horiguchi et al. (2009).

**Figure 10.**
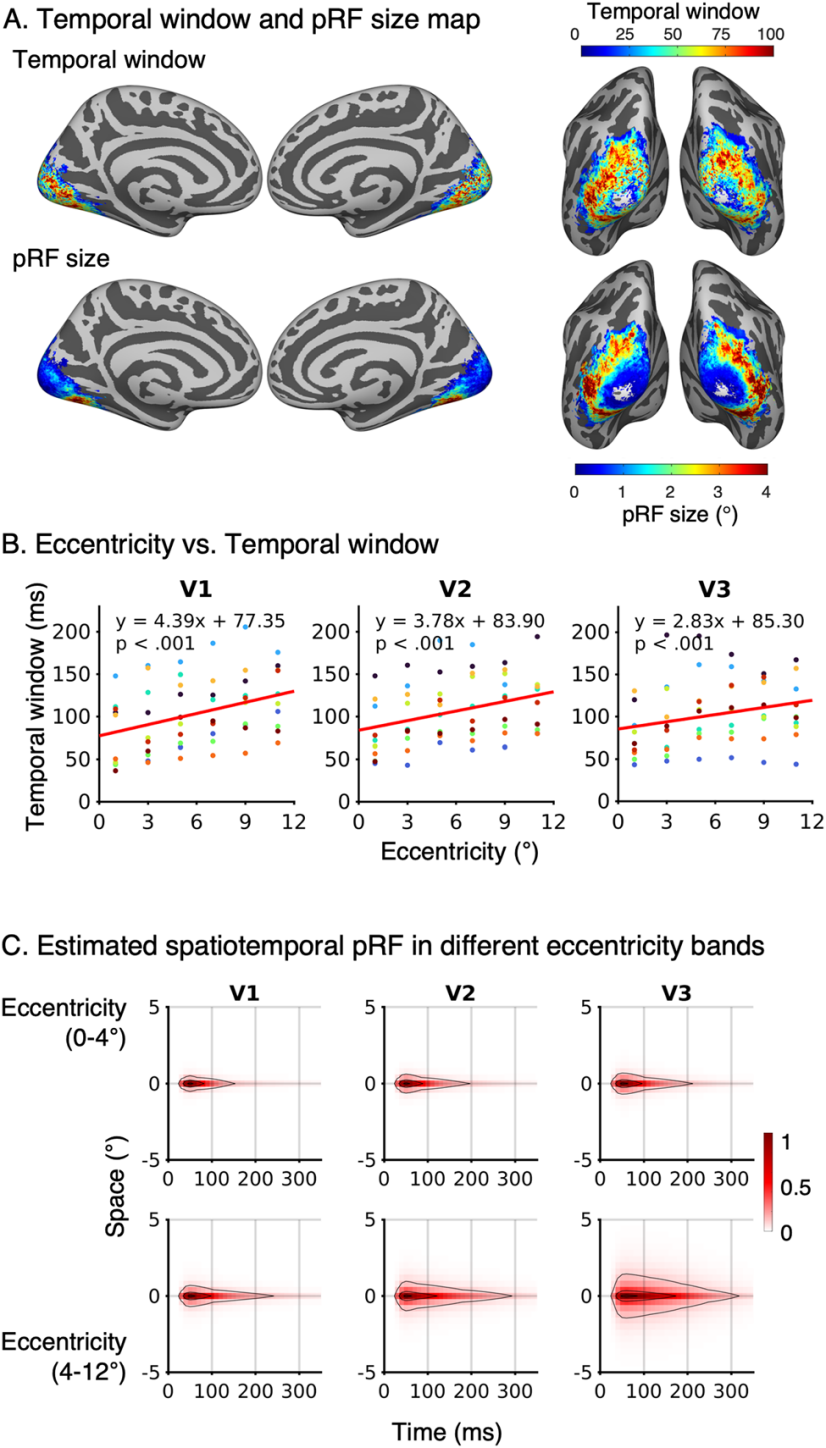
Spatiotemporal pRF properties across eccentricities in early visual cortex. ***A***, Average pRF temporal window and spatial window visualized on the FreeSurfer average cortical surface. Both hemispheres and two different viewpoints are shown (*left:* medial view, *right:* posterior view). *Top:* FWHM of average sustained temporal window estimates; *Bottom:* average pRF size. ***B***, The relationship between eccentricity and temporal window in V1, V2, and V3. The eccentricities were binned into 2° of visual angle. *Colored dots*: individual participant. *Solid line:* fitted LMM regression line. ***C***, Mean sustained spatiotemporal pRF in two different eccentricity bands (0–4° and 4–12°). Contour lines indicate 10th, 50th, and 90th percentiles.

Finally, we investigated the spatial topography of spatiotemporal receptive fields across visual cortex. Confirming our quantitative results in Figure 8*B*, qualitative examination of the maps of temporal window and pRF size reveals a topographic coupling of spatial and temporal pRF windows (Fig. 10).

To gain deeper insights, we examined this topography in early visual areas (Fig. 10*A*, left), where we observed that pRF temporal windows (Fig. 10*A*, top-left) increase along a posterior to anterior gradient and pRF sizes (Fig. 10*A*, bottom-left) also increase along the same axis. Quantitative analyses using an LMM with a fixed slope and random intercept per participant revealed a significant relationship between temporal window and eccentricity in V1, V2, V3 (Fig. 10*B*). Temporal windows were progressively larger in more peripheral eccentricities [V1: *t*_(58)_ = 8.16, *p* < 0.001; V2: *t*_(58)_ = 6.67, *p* < 0.001; V3: *t*_(58)_ = 4.19, *p* < 0.001 with corresponding beta coefficients of 4.39, 3.78, and 2.83 and across areas (intercept of 77.35, 83.90, and 85.30, respectively)]. As eccentricity also increases from posterior to anterior in early visual areas, this suggests that in V1–V3, more eccentric pRFs have larger spatial and temporal windows than foveal pRFs. Figure 10*C* visualizes the average spatiotemporal pRFs at two eccentricity bands: 0–4° and 4–12°. Indeed, in V1–V3, both pRF size and temporal window were larger in eccentricities exceeding 4° than the central 4°. The proportional increase in both spatial and temporal dimensions with eccentricity suggests that there are computational similarities in how the visual system encodes spatial and temporal information at the center and periphery.

A similar topography showing a coupling between pRF size and temporal window is also seen in the lateral surface, which contains intermediate and higher-level regions: more posterior regions have smaller spatial and shorter temporal windows than anterior regions (Fig. 10*A*, right). Within later visual areas, the eccentricity-dependent relation between pRF size and temporal windows was significant in LO (*t*_(58)_ = 2.37, *p* < 0.05), while the opposite effect was observed in VO (*t*_(58)_ = −4.87, *p* < 0.001). No significant eccentricity-dependent effects were found in V4 (*t*_(58)_ = −0.64, *p* = 0.53), TO (*t*_(58)_ = −0.88, *p* = 0.38), V3AB (*t*_(58)_ = 1.87, *p* = 0.07), and IPS (*t*_(58)_ = 0.01, *p* = 0.99).

Together these analyses reveal three organizational principles. First, both spatial and temporal windows progressive increase across the visual hierarchy. Second, within early visual cortex, both spatial and temporal windows increase with eccentricity. Third, transient responses are dominant across all three visual processing in our experiment.

## Discussion

Here, we developed a new mapping and computational framework to estimate spatiotemporal pRFs in each voxel using fMRI. This framework expands on previous studies that either estimated spatial pRFs with experiments that varied stimulus location but not timing (Dumoulin and Wandell, 2008; Kay et al., 2013; Benson et al., 2018; Aqil et al., 2021; Finzi et al., 2021) or estimated temporal characteristics with experiments that varied timing but not spatial aspects of the stimulus (Horiguchi et al., 2009; Stigliani et al., 2017, 2019; Zhou et al., 2018, 2019; Chai et al., 2019; Himmelberg and Wade, 2019; Groen et al., 2022). We find that spatiotemporal pRFs with a Gaussian spatial profile, sustained and transient temporal channels, and a compressive nonlinearity better explain fMRI responses than conventional Gaussian pRFs for spatially and temporally varying stimuli. Spatial parameters of spatiotemporal pRFs and their cortical topography replicate findings from conventional pRF methods (Wandell and Winawer, 2015). Temporal estimates and compressive nonlinearities progressively increase from earlier to later areas of visual streams. Interestingly, we find spatiotemporal coupling whereby pRFs in intermediate and later visual areas have both larger spatial and temporal windows than pRFs in earlier visual areas. Together, this spatiotemporal pRF framework pushes the temporal limits of fMRI to understand how spatiotemporal information is encoded in visual degrees and milliseconds across neural populations in human visual cortex.

### Spatiotemporal pRF framework

The spatiotemporal framework we developed serves as a testbed for systematically comparing pRF models. As all the models are tested and compared using matched HRF, optimization algorithm, and SNR of synthetic time courses, this ensures model reproducibility and validity. Moreover, each step of the data analysis pipeline is modularized to facilitate the evaluation of individual components.

Through simulation, we can identify not only the scope of model parameter interpretability given a unique visual input, number of measurements, and expected level of fMRI noise, but also evaluate different experimental paradigms. Critically, the framework enables validating that solved model parameters accurately recover the underlying spatiotemporal pRF from the stimulus and synthesized fMRI response. Applying the spatiotemporal pRF approach to synthesized fMRI data revealed that: (1) spatial parameter estimates were similar across the three pRF models tested (Spatial, DN-ST, and CST), (2) temporal parameter estimates of the CST model were more accurate than DN-ST, across experiments (Spatiotemporal/Toonotopy), and (3) temporal parameter estimates for both CST and DN-ST were more accurately estimated from the Spatiotemporal than the Toonotopy experiment.

Future research can leverage the simulator to develop optimal mapping sequences for different spatiotemporal pRF models, which would provide opportunities for better estimating model parameters (e.g., temporal parameters of DN-ST) and comparing between models. Additionally, the spatiotemporal pRF framework is flexible, enabling researchers to test other spatiotemporal models, such as ones with other spatial bases (e.g., Aqil et al., 2021), with different weightings of on- and off-transients, or with different compressive nonlinearities.

### Differences in HRF temporal parameters cannot explain neural temporal dynamics across visual areas

A key aspect of the spatiotemporal pRF approach is that it first predicts neuronal responses in milliseconds and then convolves predicted neural responses with the HRF to predict BOLD signals. This implementation allowed us to separately evaluate the effect of neural and hemodynamic responses on the resulting BOLD signal. Our data revealed that neural temporal parameters varied systematically across visual areas. In contrast, optimized HRF parameters varied more across voxels within an area than across areas (Fig. 7*B*). Critically, this variability in HRF parameters did not explain neural estimates as neural temporal parameters across areas were similar across models solved with optimized HRFs and constant HRF functions (Fig. 7*A,C*). Nonetheless, the benefit of optimizing the HRF is reducing between-voxel variability as indicated by Prince et al. (2022). Notably, this spatiotemporal pRF approach is not only necessary for predicting the observed BOLD responses—especially to brief stimuli that are separate by short interstimulus intervals—but also is more parsimonious than an approach using different HRFs for fast and slow stimuli (Lewis et al., 2016; Polimeni and Lewis, 2021).

### Comparing human temporal pRFs and neuronal temporal receptive fields

We found substantial variability in temporal parameters within an area as well as overlap in temporal parameters across areas (Fig. 7*A*). In particular, V1, V2, and V3 contain pRFs with a large range of temporal windows that also vary with eccentricity (Fig. 10). The temporal overlap between areas (1) may indicate some level of parallel temporal processing across visual areas and (2) could be related to the underlying neural architecture as anatomical connections in the visual system are not strictly feedforward from one area to the next due to feedback and recurrent connections (Felleman and Van Essen, 1991; Nowak and Bullier, 1997; Lamme and Roelfsema, 2000).

**Figure 11.**
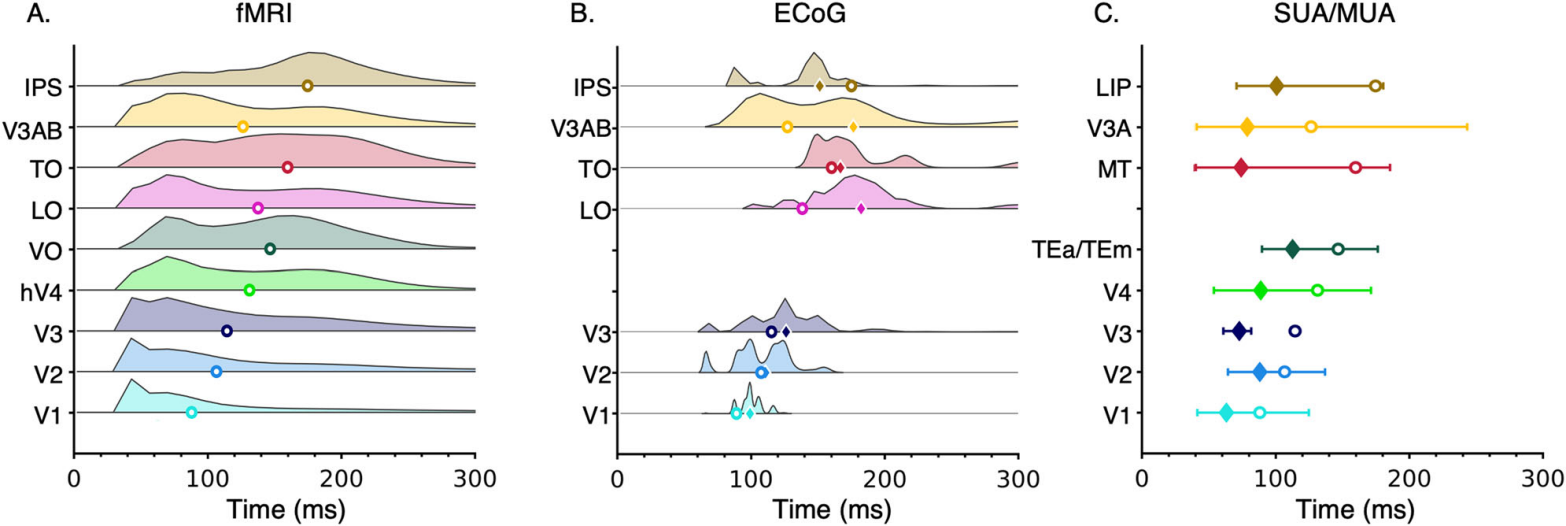
Comparison of estimates of temporal latency in this study to ECoG in humans and electrophysiology in nonhuman primates. In all panels, each row illustrates data from a visual area; *x*-axis: time. ***A***, Distribution of temporal latency estimated from the current study using fMRI. *Open dot*: median of time-to-peak latency. ***B***, Latency distributions replotted from a human ECoG study (Groen et al., 2022; https://openneuro.org/datasets/ds004194). *Diamond*: median of time-to-peak latency. *Open circle:* median fMRI latency. ***C***, Temporal latency ranges from nonhuman primates measured using electrophysiology; data are aggregated over multiple studies. The central diamonds indicate the average median or mean latency values reported. The whiskers represent the average of minimum/maximum or 10th/90th percentile latency values. *Open circle:* median fMRI latency. Data are from the following studies: *V1* (Raiguel et al., 1989; Knierim and van Essen, 1992; Maunsell and Gibson, 1992; Celebrini et al., 1993; Vogels and Orban, 1994; Nowak et al., 1995; Schmolesky et al., 1998; Bair et al., 2002), *V2* (Raiguel et al., 1989; Nowak et al., 1995; Schmolesky et al., 1998), *V3* (Schmolesky et al., 1998), *V4* (Schmolesky et al., 1998; Chang et al., 2014; Zamarashkina et al., 2020), *TEa/TEm* (Baylis et al., 1987), *MT* (Raiguel et al., 1989, 1999; Schmolesky et al., 1998; Bair et al., 2002; Nakhla et al., 2021), *V3A* (Nakhla et al., 2021), and *LIP* (Barash et al., 1991).

Given this variability and that we estimate temporal parameters from fMRI responses, it is interesting to compare time-to-peak latencies derived from the CST model to those from measurements that afford high temporal resolution [electrocorticography (ECoG) in humans; single and multiunit electrophysiology in nonhuman primates]. While latency estimates from fMRI were more variable than either ECoG or electrophysiology, we find strikingly comparable time-to-peak latencies across measurement modalities (Fig. 11). This similarity is despite substantial methodological differences in experimental procedures, number of measurements, stimuli, species, and usage of anesthetics. Although more validations are needed, the larger variability in the fMRI estimates is somewhat expected, as fMRI enables broader coverage of each visual area, effectively sampling from a larger neural population than other modalities. There are three main similarities across our data, ECoG data, and electrophysiology measurements (Fig. 11). First, earlier visual areas display shorter response latencies than later visual areas. Second, within each area, there is variability in temporal latencies of neurons and voxels, reflecting heterogeneous response properties (Saul and Humphrey, 1990; Henry et al., 2020). Third, across areas, there is a significant degree of overlap of response latencies, suggesting some level of parallel processing in visual cortex (Nowak and Bullier, 1997; Schmolesky et al., 1998).

Quantitatively, our estimates of time-to-peak latencies in V1–V3 (40–120 ms) are within the range of ECoG: 60–80 ms (Martin et al., 2019) to 100–120 ms (Zhou et al., 2019; Groen et al., 2022; Fig. 11*B*). Likewise, our estimates of time-to-peak latencies in intermediate (V3AB, LO) and higher-level areas (TO, IPS), ranged from 70 to 200 ms, consistent with ECoG data that ranged from 80–150 ms (Martin et al., 2019) to 170–200 ms (Groen et al., 2022). However, we note that mean estimates of temporal latencies in humans are consistently slower than in macaques by at least 18 ms (Fig. 11*C*), which may reflect a species difference in temporal processing.

### Hierarchical and parallel processing in visual cortex

Our data reveal that spatiotemporal pRF properties vary across the processing hierarchy as well as between later visual areas in different streams. Consistent with the first hypothesis (Zhou et al., 2018), we found progressive increases in pRF spatial and temporal windows as well as in compressive nonlinearities from earlier to later visual areas. This hierarchical structure of spatiotemporal processing of visual inputs may be achieved via accumulated spatiotemporal pooling across a feedforward neural architecture (Felleman and Van Essen, 1991; Lennie, 1998; Yamins et al., 2014). It is interesting that spatiotemporal characteristics of intermediate visual areas (V3AB, LO, hV4) were similar across streams, but those of later visual regions (IPS, TO, VO) diverged across streams, with IPS having the longest temporal window and TO having the largest spatial window. These findings raise the intriguing hypothesis that the subsequent regions in each of these processing streams would show even more divergence. For example, IPS2-4 located in the dorsal stream may have even more divergent spatiotemporal properties than pFus-faces and mFus-faces located in the ventral stream. Future experiments can investigate if and how spatiotemporal pRFs differ across streams in other spatial and temporal aspects including eccentricity-dependent processing of speed (Carrasco et al., 2003), motion more broadly (Traschütz et al., 2012), temporal contrast sensitivity (Himmelberg and Wade, 2019), temporal color sensitivity (Seidemann et al., 1999; Conway and Livingstone, 2006; Gentile et al., 2023), and visual capacity (Kupers et al., 2023).

## Conclusions

In summary, the spatiotemporal pRF framework offers new avenues to evaluate spatiotemporal processing at the resolution of visual degrees and milliseconds, which was not thought to be possible with fMRI due to the slow nature of hemodynamic responses. This opens exciting new possibilities for modeling and measuring spatiotemporal processing across other brain systems such as the auditory system (deCharms et al., 1998; Theunissen et al., 2001; Stephens et al., 2013), somatosensory system (Sripati et al., 2006; Reed et al., 2010), as well as examining the sequence and time dependence of cognitive processing broadly including working memory (Zylberberg and Strowbridge, 2017) and decision making (Gold and Shadlen, 2007; Louie et al., 2013).
